# Adverse effects of tyrosine kinase inhibitors in cancer therapy: pathophysiology, mechanisms and clinical management

**DOI:** 10.1038/s41392-023-01469-6

**Published:** 2023-07-07

**Authors:** Sunitha Shyam Sunder, Umesh C. Sharma, Saraswati Pokharel

**Affiliations:** 1 Cardio-Oncology Research Group, Department of Pathology and Laboratory Medicine, Roswell Park Comprehensive Cancer Center, Buffalo, NY USA; 2 Division of Cardiovascular Medicine, Jacob’s School of Medicine and Biomedical Sciences, University at Buffalo, Buffalo, NY USA

**Keywords:** Cardiology, Oncology, Pathogenesis, Gastrointestinal diseases

## Abstract

Since their invention in the early 2000s, tyrosine kinase inhibitors (TKIs) have gained prominence as the most effective pathway-directed anti-cancer agents. TKIs have shown significant utility in the treatment of multiple hematological malignancies and solid tumors, including chronic myelogenous leukemia, non-small cell lung cancers, gastrointestinal stromal tumors, and HER2-positive breast cancers. Given their widespread applications, an increasing frequency of TKI-induced adverse effects has been reported. Although TKIs are known to affect multiple organs in the body including the lungs, liver, gastrointestinal tract, kidneys, thyroid, blood, and skin, cardiac involvement accounts for some of the most serious complications. The most frequently reported cardiovascular side effects range from hypertension, atrial fibrillation, reduced cardiac function, and heart failure to sudden death. The potential mechanisms of these side effects are unclear, leading to critical knowledge gaps in the development of effective therapy and treatment guidelines. There are limited data to infer the best clinical approaches for the early detection and therapeutic modulation of TKI-induced side effects, and universal consensus regarding various management guidelines is yet to be reached. In this *state-of-the-art* review, we examine multiple pre-clinical and clinical studies and curate evidence on the pathophysiology, mechanisms, and clinical management of these adverse reactions. We expect that this review will provide researchers and allied healthcare providers with the most up-to-date information on the pathophysiology, natural history, risk stratification, and management of emerging TKI-induced side effects in cancer patients.

## Introduction

Innovations in the field of cancer therapies have led to the effective management of different cancers, which were previously considered to be incurable. Although these treatments have dramatically changed the natural course of many cancers, they may result in cardiac and extracardiac complications, which can be manifested either during therapy or after the completion of treatment. Both traditional therapies like anthracyclines, alkylating agents, and antimetabolites,^1^ and newer cancer therapies such as pathway-directed targeted therapies, and immunotherapies^2,3^ can have cardiovascular (CV) and metabolic sequelae, resulting in heart failure (HF), coronary artery disease (CAD), myocarditis, arrhythmias, and vascular and metabolic disturbances.

In the human genome, ninety tyrosine kinases have been identified, including fifty-six receptor tyrosine kinases and thirty-two cellular tyrosine kinases.^4^ Tyrosine kinase inhibitors (TKIs) used as targeted therapies are designed to perturb the cellular pathways that regulate malignant cell growth.^1^ TKI can be generally categorized into small molecules and macromolecules (e.g., monoclonal antibodies, polypeptides, antibody–drug conjugates, and nucleic acids).^5,6^ TKIs target signaling pathways involving the receptor tyrosine kinase and/or intracellular kinases that regulate cellular proliferation and tumor angiogenesis.^2,6,7^ The selectivity of binding of various TKIs to their targets influences the potency, mechanism of action, selectivity, and safety profile of these agents.^8^ Only select TKIs exhibit selectivity to specific protein kinases whereas most of the TKIs inhibit multiple kinases (~10–100) leading to the increased risk of toxicities.^9^ For instance, prior studies reported a ~ 5% rate of discontinuation of multi-kinase inhibitor (MKIs), imatinib therapy due to TKI-associated adverse effects.^10^ Toxicity can be attributed to both *on-target* effects through excessive inhibition of the intended TK function; and/or *off-target* effects resulting from the simultaneous inhibition of multiple other kinases due to limited selectivity.^8,11,12^ *On-target effects* have been implicated in the causation of adverse reactions such as hypertension (HTN), hypothyroidism, skin reactions, and proteinuria.^13^ However, it is important to identify the close relationship between the effectiveness of therapy and the occurrence of toxic reactions. The side effect profile is frequently used as a monitoring tool to identify the desired outcomes of cancer therapy.^14^ However, a positive correlation has been identified between the number of kinases inhibited and the extent of their toxic potential.^15^

Several TKIs have been approved by the US Food and Drug Administration (Table 1). TKI-associated side effects can be mild to life-threatening affecting various organ systems, and in some cases, severe adverse effects require premature discontinuation of life-saving cancer therapies. Cardiotoxicity has been asserted as a notable effect of several agents including TKIs. Although TKIs are often used as therapeutics in oncology, it should also be noted that TKIs utilized in other fields of pharmacotherapy, for instance, TKIs used as antipsychotics, can also induce cardiotoxicity.^16^ TKI-related cardiovascular dysfunction (TRCD) can have serious consequences ultimately leading to premature treatment cessation or interruption. In this review, we provide a comprehensive summary of commonly reported cardiac and extracardiac side effects of TKIs used as therapy in oncology, with a specific focus on the early detection and clinical management of these side effects. Special emphasis is given to the CV side effects due to the complexity of screening and management of these complications.

**Table 1:** Small molecule TKIs approved by US FDA for use in cancer therapy, their indications, and common adverse effects

Receptor targets	Drugs		Applications	Cardiovascular Adverse Effects	Extra-Cardiac Adverse Effects
**ALK family** (Anaplastic lymphoma kinase)	First-generation: **Crizotinib** (Type Ia c**-MET** inhibitor)		ALK+, ROS1 + NSCLC	Arrhythmia, QT Prolongation, Bradycardia	Visual disturbances (flashes, light columns, blurred vision), Neutropenia
	Second-generation:	**Ceritinib**	ALK + NSCLC as first-line treatment or after crizotinib resistance	QT Prolongation Arrhythmia, MI, Bradycardia	GI disorders
		**Alectinib**	ALK + NSCLC	QT Prolongation, Arrhythmias	Anemia
		**Brigatinib**	ALK+ NSCLC after crizotinib	Arrhythmia, Bradycardia, HTN	ILD
		**Ensartinib**	ALK+ NSCLC	–	Rash, Elevated transaminase levels (AST/ALT), Pruritus, GI disorders, Edema, Anemia, Increased levels of blood ALP blood creatinine, GGT, Increased bilirubin level, Increased CPK level, and Hyponatremia
	Third-generation:	**Lorlatinib**	ALK + NSCLC	MI, PR interval prolongation, AV block	Fatigue, Increased ALT, Cough, Anemia, Decreased neutrophil count, Mental disorders, Mood, speech, and sleep disorders
		**Entrectinib**	ROS1 + NSCLC; solid tumors with NTRK fusion proteins	QT Prolongation	Dysgeusia, Dizziness, Weight Gain, Paresthesia, Fatigue, GI Disorders, Peripheral edema, Myalgia, Anemia, Increased Blood Creatinine, and Arthralgia.
	**Repotrectinib (**Next- Generation **ROS1/TRK/ALK Inhibitor**		ROS1+ advanced NSCLC	–	Dizziness
**BCR–ABL family** [Fusion protein of ABL1 (Abelson murine leukemia viral oncogene homolog 1) and Breakpoint cluster region protein (BCR)-cytoplasmic fusion tyrosine kinase]	First-generation: **Imatinib** (also targets **VEGFR** and **PDGFR)**		Ph+ CML or ALL, CEL, DFSP, HES, GIST, MDS/MDP	HF, LVD	Nausea, Vomiting, Diarrhea, Edema, Myelosuppression, Immunosuppression, Fatigue, Insomnia, Depression, Dizziness, URTI, influenza, Pyrexia, Cough, Abdominal pain, Myalgia, Arthralgia, Skin rash, Hemorrhage Rare association with appendiceal carcinoma
	Second-generation:	**Nilotinib**	Ph+ ALL	MI, QT prolongation, HTN	Myelosuppression, Hyperbilirubinemia, Pancreatitis, Fatigue, Headache, Nausea, Vomiting, Diarrhea, Constipation, Skin rash, Pruritis, Edema
		**Dasatinib** [also targets **Src (**cytoplasmic tyrosine kinase)]	Ph+ CML and ALL	Pleural/Pericardial Effusion, QT prolongation, HF, LVD, MI	Myelosuppression, Panniculitis, Bleeding, Fatigue, Headache, Dyspnea, Infection, Fluid retention, Abdominal pain, Nausea, Diarrhea, Edema Myalgia, Arthralgia, Skin rash, Hemorrhage
		**Bosutinib** (also targets **Src)**	CML resistant or intolerant to prior TKI therapy	QT prolongation, PHTN, Pericardial Effusion	Myelosuppression, Fatigue, Headache, Dyspnea, Cough, Pyrexia, GI disorders, Edema, Increased ALT, Arthralgia, Skin rash
	Third-generation: **Ponatinib**		Ph+ CML or ALL	HTN, HF, Arrhythmias	Myelosuppression, Fatigue, Headache, Dyspnea, Pyrexia, Pancreatitis, Pyrexia, Abdominal pain, Nausea, Vomiting, Diarrhea, Constipation, Increased ALT, Myalgia, Arthralgia, Skin rash, Dry skin, Hepatotoxicity, Liver failure, and Death
**B-Raf family** (B-rapid accelerating fibrosarcoma/v-raf murine sarcoma viral oncogene homolog B1)	**Vemurafenib**		Melanoma with BRAFV600E mutation, and ECD	LVD, QT prolongation, AF	Photosensitivity, Skin rash, Increased LFTs, Arthralgia, Nausea, Fatigue, Edema, Cutaneous squamous- cell carcinoma, Pruritus, Palmar–plantar dysesthesia
	**Dabrafenib**		Melanoma and NSCLC with BRAF mutations	LVD	Fever, Neutropenia, Arthralgia, Fatigue, Headache, Peripheral edema
	**Encorafenib**		BRAF V600E/K mutant melanoma with binimetinib	QT prolongation, LVD	Anemia, Transient Bell’s palsy, Myalgia, Nausea, Palmoplantar erythrodysesthesia, Arthralgia, Alopecia, Hyperkeratosis.
**BTK family (**Bruton’s tyrosine kinase)	**Ibrutinib**		MCL, CLL, WM, GVD, MZL	Arrhythmia, HTN	Hemorrhage, Cytopenias Diarrhea, Increased risk of Infections, Arthralgia, Fatigue, Muscle Spasms or Myalgias, Pyrexia, Skin Rash, Headaches
	**Acalabrutinib**		MCL	Arrhythmia	Hemorrhage, Headache, Diarrhea, Fatigue, Myalgias, Cough, Neutropenia, Nausea, Skin Rash, and Infections.
	**Zanubrutinib**		Refractory MCL and WM	Arrhythmia	Neutropenia, Increased Risk of Infections
**c-MET family** (Hepatocyte Growth Factor (HGF)/Mesenchymal al-Epithelial Transition Factor Receptor)	**Capmatinib** (Type Ib inhibitor)		NSCLC with *MET*ex1 4 (MET exon 14 skipping mutation)	–	ILD/Pneumonitis, Peripheral Edema, Fatigue, GI Disorders, Grade 3 hepatotoxicity, Elevated Creatinine clearance, Photosensitivity
	**Tepotinib** (Type Ib inhibitors)		NSCLC) with METex14	–	Peripheral edema, Nausea, Diarrhea, Increased blood creatinine, Upper abdominal pain, Hypoalbuminemia, Increased ALT/AST, amylase and lipase levels, Asthenia, Decreased appetite, Pleural effusion, Alopecia, Fatigue, General edema
**EGFR/ERBB family** ErbB1 [EGFR (human epidermal growth factor receptor) or HER1 (erythroblastic leukemia viral oncogene B), ErbB2 (HER2), ErbB3 (HER3), and ErbB4 (HER4) [EGFR family receptor tyrosine kinase]	First-generation (Competitive and Reversible)	**Gefitinib**	NSCLC	MI	Skin rash, Diarrhea, Nausea, ILD, Dry Skin, Pruritus, Stomatitis, Anorexia
		**Erlotinib**	NSCLC and Pancreatic Cancer	Edema	Skin rash, Diarrhea, Fatigue, Appetite loss, Nausea, ILD, Hematologic, Alopecia, Arthralgia, Neuropathy
		**Lapatinib** (reversible dual inhibitor- EGFR and HER2)	Breast Cancer	HF, LVD, QTProlongation	Skin rash, Diarrhea
	Second-generation (Covalent and Irreversible)	**Vandetanib** (also targets **Src/VEGFR 2/RET**)	MTC	Asymptomatic QT prolongation, Torsades de pointes, Arrhythmia, HF, HTN	Rash, Diarrhea, Proteinuria
		**Afatinib**	NSCLC	HTN	Severe Diarrhea, Rash-Acne, Stomatitis, Paronychia, Dry Skin, Appetite loss
		**Dacomitinib**	*EGFR*-mutated NSCLC	–	Diarrhea, Paronychia, Rash-Acne, Stomatitis, Dry Skin, Appetite loss, Weight Loss, Alopecia, Cough, Hemorrhoids, Wound, Back pain, Headache
		**Neratinib**	HER2+ breast cancer	Low rates of symptomatic decline in LVEF and QT prolongation	Diarrhea, GI disorders, Fatigue, Headache
	Third-generation (Irreversible): **Osimertinib**		NSCLC	QT prolongation, LVD, HF, Arrhythmia, MI, Pericardial Effusion	Diarrhea, Rash, Dry Skin, Paronychia, Stomatitis, Fatigue
	**Mobocertinib** (Irreversible)		*EGFR* exon 20 insertions (*EGFR*ex20i ns) mutation-driven NSCLC	–	GI Disorders, Rash, Dry skin, Stomatitis, Fatigue, Maculopapular Rash, Paronychia, Anemia, Dermatitis acneiform, Increased lipase, Pruritus
	**Pyrotinib (**Irreversible **dual pan-ErbB** receptor inhibitor)		HER2- positive		**Diarrhea, Hand-foot syndrome**, Leukopenia, Neutropenia, GI disorders, Increased ALT, Anemia, Asthenia
**FGFR family** (fibroblast growth factor receptor)	**Erdafitinib**		Urothelial carcinoma	MI	Ocular disorders (central retinopathy), Hyperphosphatemia, Embryo-fetal toxicity, Fatigue, Dysgeusia, Paronychia, Alopecia, HFSR, Xerosis
**Flt3 family** (Fms-like tyrosine kinase 3)	**Gilteritinib**		AML with FLT3 mutation5	QT prolongation	Arthralgia/myalgia, Dizziness, Dyspnea, Edema, Fatigue, Noninfectious diarrhea, Pneumonia, Rash, Transaminitis
	**Midostaurin**		ALL Flt3 mutation+	HTN, Pericardial Effusion	Nausea, Vomiting, Diarrhea, Fatigue, Headaches
**JAK family (**Janus Kinase)	**Ruxolitinib**		MF and PV	MI, Venous thromboembolism	Myelosuppression, Hematological toxicity particularly thrombocytopenia, anemia - dose-limiting toxicity, ecchymosis, Dizziness, and Fatigue
	**Fedratinib**		Primary or secondary myelofibrosis	HF, Cardiogenic Shock	Anemia, GI symptoms, Increased levels of liver transaminases, serum creatinine, and pancreatic enzymes, Encephalopathy
**MAPKK family/ MEK ½** (Mitogen-activated protein kinase kinase)	**Trametinib**		Melanoma (2013) and NSCLC (2017) with BRAF mutations	LVD	Rash, Diarrhea, Central Serous Retinopathy, Papulopustular Exanthema, Peripheral edema,
	**Binimetinib**		BRAF V600E/Kmelanoma with encorafenib	QT prolongation, LVD	Papulopustular rash, Central Serous-like Retinopathy, GI Disorders
	**Cobimetinib**		Melanoma with BRAF V600E/K mutations with vemurafenib	LVD, QT prolongation	Rash, Fatigue, Edema, GI Disorders
**NTRK family** (Neurotrophic Tyrosine Receptor Kinase)	**Larotrectinib**		Solid tumors with NTRK gene fusion proteins	–	Anemia, Increased AST/ALT, Weight increase, Decreased neutrophil count, Fatigue, Cough
**PDGFR family** (PDGFR α/β, KIT [CD117, stem cell factor receptor, Colonial stimulating factor-1 receptor (CSF1R), the stem cell growth factor receptor (SCGFR), FLK2/FLK3][Receptor tyrosine kinase] & **VEGFR family** (VEGFR1, VEGFR2, VEGFR3) [Receptor tyrosine kinase]	**Sorafenib** (also targets **CDK, B-RAF, KIT, FLT-3, RET, c-MET**)		RCC, DTC, and HCC	HTN, ACS, HF, Arterial thromboembolism, QT prolongation	Skin rash, Hemorrhage, HFSR, Mucositis, Hypothyroidism, Fatigue, Renal toxicities, including proteinuria and acute renal failure, Dyspnea, Diarrhea
	**Sunitinib** (also targets **Src)**		HES, GIST, MDS/MDP	HTN, MI, HF, Reduced LVEF, LVD, Arterial thrombosis, QT prolongation, Torsades de pointes	Hemorrhage, Hypothyroidism, Adrenal Dysfunction
	**Ponatinib** (also targets **Src and FGFR)**		Ph+ CML or ALL	HF, Arrhythmia, HTN, Cardiomyopathy, Vascular occlusion, Arterial, and Venous thrombosis	Rash, Abdominal pain, Nausea, Constipation, Headaches, Dry skin, Fatigue, Fever, Myalgia. Dyspnea, Arthralgia, Increased ALT/ lipase, Pancreatitis and amylase increase, Hematologic adverse effects (thrombocytopenia > neutropenia > anemia), Hepatotoxicity, Liver failure, and Death
	**Axitinib** (also targets **KIT**)		RCC	HTN, HF, Arterial Thrombosis. Hypotension	Fatigue, Nausea, Diarrhea, Vomiting, Headache, Hemoptysis, Stomatitis, Erythema, Anorexia, Limb Pain, Arthralgia, Myalgia Hand-Foot Syndrome, Dyspnea, Dehydration
	**Regorafenib**		CRC, GIST	MI, HTN, Arterial thrombosis	HFSR reactions, Diarrhea, Fatigue, Anemia, Thrombocytopenia, Proteinuria
	**Pazopanib**		RCC, STS	LVD, HTN, HF, Arterial thrombosis, Cardiomyopathy, QT Prolongation, Torsades de pointes	Fatigue, GI disorders, Elevated ALT, Neutropenia, Leukopenia, Lymphocytopenia, Anemia, Asthenia
	**Lenvatinib** (also targets **Src)**		DTC	QT Prolongation, LVD, HF, HTN	Hypothyroidism, Diarrhea, Fatigue, Decreased appetite
	**Cabozantinib** (Type II **c-MET** inhibitor) (also targets **Src** and**)**		Metastatic MTC, advanced RCC, and HCC	HTN, Arterial thrombosis	Palmar–plantar erythrodysesthesia, GI disorders, Fatigue, Stomatitis.
	**Praseltinib** (also inhibits **RET,** **DDR1, TRKC, FLT3, JAK1–2, TRKA, VEGFR2, PDGFRb, and FGFR1)**		Metastatic *RET* fusion + NSCLC Advanced or metastatic *RET*-mutant MTC Advanced or metastatic *RET* fusion + thyroid cancer	HTN	Rash, Anemia, Cough, Fatigue, Pyrexia, GI disorders, Edema, Musculoskeletal pain, Decreased WBCs, Hyperphosphatemia, Increased AST/ALT, Pneumonitis, Headache, Peripheral neuropathy, Dizziness, Dysgeusia
**RET family**	**Alectinib**		ALK + NSCLC	QT Prolongation, Bradycardia	GI disorders, Myalgia, Peripheral edema, Elevated liver enzymes, Elevated blood bilirubin, Anemia
	**Selpercatinib**		Metastatic RET fusion-positive NSCLC, advanced or metastatic *RET*-mutant MTC	HTN, QT prolongation,	Hepatotoxicity, Hemorrhagic events, Hypersensitivity, risk of impaired wound healing, and Embryo-fetal toxicity.

*ACS* Acute Coronary Syndrome, *AF* Atrial Fibrillation, *ALL* Acute lymphocytic leukemia, *ALP*, Alkaline Phosphatase, *ALT* Alanine Transaminase, *AST* Aspartate Transaminase, *AV* Atrioventricular, *CEL* Chronic Eosinophilic Leukemia, *CLL* Chronic lymphocytic leukemia, *CML*, Chronic myeloid leukemia, *CPK* Creatine Phosphokinase, *CRC* Colorectal cancer, *DFSP* Dermatofibrosarcoma protuberans, *DTC* Differentiated thyroid cancer, *ECD* Erdheim-Chester Disease, *GGT* Gamma Glutamyl Transference, *GI* Gastrointestinal, *GIST* Gastrointestinal Stromal Tumor, *GVD* graft versus host disease, *HCC* Hepatic Cell Carcinoma, *HES* Hyper eosinophilic syndrome, *HF* Heart Failure, *HFSR* hand–foot skin reaction, *HTN* Hypertension, *ILD* Interstitial Lung Disease, *LFT* Liver function test, *LVD* Left Ventricular Dysfunction, *LVEF* Left ventricular ejection fraction, *MCL* Mantle cell lymphoma, *MDP* Myeloproliferative Disorders, *MDS* Myelodysplastic syndromes, *MF* Myelofibrosis, *MI* Myocardial Infarction, *MTC* Medullary thyroid cancer, *MZL* Marginal zone lymphoma, *NSCLC* Non-Small Cell Lung Cancer, *PDGFR* Platelet-Derived Growth Factor Receptor, Ph+ Philadelphia chromosome, *PHT* Pulmonary hypertension, *PV* Polycythemia vera, *RCC* Renal Cell Carcinoma, *ROS* Reactive Oxygen Species, *STS* Soft tissue sarcoma, *TKI* Tyrosine Kinase Inhibitor, *URTI* Upper Respiratory Tract Infection, *VEGFR* Vascular Endothelial Growth Factor Receptor, *WBC* White Blood cell, *WM* Waldenstrom macroglobulinemia

## Cardiovascular adverse effects

Seven cardiotoxic cancer therapy classes (including four classes of targeted therapies) are proposed based on the range of CV toxicities.^17^ CV complications are reported in both single-targeted and multi-targeted TKIs. The list of commonly used and novel TKIs with common CV adverse effects is listed in Table 2. In the heart, TKIs can adversely affect vascular endothelial cells and cardiac post-mitotic cells, predominantly cardiomyocytes (CMs).^18,19^ TKIs, specifically those with multikinase activities, affect several signal transduction pathways, of which the most affected ones include cardiotrophin and its receptor gp130, epidermal growth factor receptor (EGFR, ErbB2, or HER2), phosphoinositide 3-kinase (PI3K), AMP-activated protein kinase (AMPK), ubiquitin proteasomal system, and lysosomal autophagy pathways.^20^ Similarly, vascular endothelial growth factors (VEGF) involved in angiogenesis, microvascular function, and myocardial perfusion are often implicated in the development of TKI-induced CV target-organ damage.^21^

**Table 2:** Novel TKIs used in cancer therapy and their common cardiovascular side effects

Drug Class and Name	Molecular target	Common cardiovascular complications
Her2 inhibitors (trastuzumab, pertuzumab, lapatinib, neratinib)	erbB2/HER2	A decline in LVEF, Congestive heart failure
VEGF signaling pathway inhibitors (bevacizumab, ramucirumab, aflibercept)	VEGF-A	Hypertension, Cardiomyopathy
TKI with anti-VEGF activities (sunitinib, sorafenib, pazopanib, axitinib, vandetanib, regorafinib, cabozatenib, lenvatinib	VEGFR and other kinases e.g. PDGFR	Hypertension
Multi-targeted TKIs (imatinib, dasatinib, ponatinib, nilotinib)	Abl, abl mutant (except T315I), EGFR, PDGFR, SRC, KIT, BRAF, DDR1, DDR2, Ephrin receptor	Vascular events, QTc prolongation, Pulmonary hypertension (with dasatinib)
ALK inhibitors (crizotinib, ceritinib)	ALK	QTc prolongation, Bradycardia
BTK inhibitors (ibrutinib)	BTK	Atrial fibrillation, Ventricular arrhythmia
MEK inhibitors(trametinib)	MEK1/MEK2	Cardiomyopathy

*ALK* Anaplastic lymphoma kinase, *BRAF* B-rapid accelerating fibrosarcoma, *BTK* Bruton’s Tyrosine Kinase, *EGFR* Epidermal Derived Growth Factor Receptor, *LVEF* Left ventricular ejection fraction, *MEK* Mitogen-activated protein kinase kinase, *PDGFR* Platelet-Derived Growth Factor Receptor, *TKI* Tyrosine kinase inhibitors, *VEGFR* Vascular Endothelial Growth Factor Receptor

Mechanisms such as reactive oxygen species (ROS), accumulation of drug metabolites disrupting the structure and function of sarcomeres, and mitochondrial biogenesis leading to vacuole formation, contractile element disarray, disruption of mitochondrial transport chains leading to activation of various cell death pathways, including ferroptosis, pyroptosis, and necrosis have been implicated.^11,16,22^ Specifically, apoptosis can be induced through the activation of extrinsic (*cell surface death receptors*) and intrinsic [*mitochondria and the endoplasmic reticulum (ER)]* pathways.^23^ Necroptosis and ferroptosis are caspase-independent and iron-dependent processes, respectively.^24,25^ Necroptosis can be induced mainly through the stimulation of various death receptors and Toll-like receptors.^23^

## Major TKI related cardiovascular events

### Myocardial dysfunction and heart failure

In various clinical studies, TKIs were reported to be associated with the highest relative risk (RR 5.6) for *high-grade* cardiotoxicity. A meta-analysis of clinical trials in 10,647 patients with a wide range of malignancies treated with TKIs (axitinib, pazopanib, sorafenib, sunitinib, and vandetanib) showed a combined incidence of asymptomatic left ventricular dysfunction (LVD) of 2.4% with no differences between more specific TKIs and MKIs.^26^ Among the TKIs, HER2 molecular-targeted therapies such as lapatinib, vascular endothelial growth factor inhibitors (VEGFIs), and BCR-ABL TKIs were associated with a higher risk of HF or LVD.^27,28^ Similarly, VEGFIs are known to induce chronic progressive microvascular changes which reduce the myocardial capillary network (rarefaction) leading to myocardial hypoperfusion and impaired contractility.^29^

Traditionally, the term type I and type II cardiotoxicity are used to describe the pattern of cancer therapy-induced cardiotoxicity.^30,31^ Anthracyclines are the prototype drugs in type I cardiotoxicity and are responsible for acute as well as progressive and cumulative myocellular injury or loss.^32–34^ In contrast, trastuzumab and TKIs are classic examples of type II cardiotoxicity characterized by late-onset, non-progressive, and reversible features (Table 3).^22^ However, the growing evidence suggests that this categorization is both incomplete and fundamentally incorrect. A study involving 45,000 breast cancer patients treated with anthracycline, trastuzumab, or the combination revealed that trastuzumab caused more long-term damage than anthracyclines in real-world patients with breast cancer. These findings debunk the notion that trastuzumab-treated patients aren’t susceptible to “sequential stress-induced cardiomyopathy” in the same fashion as anthracycline-treated patients.^35^ Several other TKIs have been known to cause irreversible cardiac changes.

**Table 3:** Types of cardiotoxicity

Type I Cardiotoxicity	Type II Cardiotoxicity
• Early Onset	• **Late** Onset
• Myocardial damage	• Myocardial **Dysfunction**
• Permanent / Irreversible	• **Reversible** in nature
• Dose-dependent effects	• Cumulative Dose-**independent** effects
• Greater association with Cardiac Dysfunction and Clinical HF	• **Increased loss of contractility** and less myocyte death
• Typically, with Anthracyclines (Doxorubicin), Alkylating Agents Taxanes, Topoisomerase Inhibitors, Antimetabolites	• Typically, with Trastuzumab, Bevacizumab, and other Tyrosine Kinase Inhibitors, Immunomodulatory drugs, and Proteasome inhibitors but patients may develop type I cardiotoxicity in the long run

From the mechanistic perspective, CM death was reported in cardiac-specific VEGF knockout mice, and also in mice treated with VEGFI.^36^ Similar effects were observed in mice treated with platelet-derived growth factor inhibitors (PDGFI), further highlighting the effects of VEGF in the development of microvascular dysfunction through PDGF inhibition.^37^ Importantly, VEGFI-induced LVD is predisposed by previous or concurrent cardiotoxic chemotherapies creating a *vulnerable myocardium*.^26^ Sunitinib and sorafenib impair the angiogenic response necessary to overcome the effects of HTN-induced pressure overload to the heart, thus resulting in an increased incidence of cardiac dysfunction and HF.^2^ Sunitinib is also reported to inhibit platelet-derived growth factor receptors (PDGFR) resulting in decreased myocardial pericytes and microvascular density.^38^ Similar findings were reported in pazopanib-treated patients with some unique case reports of the apical ballooning syndrome and rapidly progressive fulminant HF.^39^

The neuregulin-HER pathway (NRG-1/HER-4/HER-2 axis) inhibition is responsible for the anti-HER-2 TKI-related HF pathophysiology. Based on the cardioprotective role of neuregulin-1 (NRG-1), clinical trials assessing its safety and efficacy in HF are currently underway.^40^ Cardiotoxicity with anti-HER2 TKIs was seen at a higher rate in metastatic trials compared to adjuvant trials.^28^ In a meta-analysis evaluating the cardiac adverse events of anti-HER2 drug lapatinib-treated patients, the overall incidence associated with breast cancer was found to be higher than in other cancers (3% vs 2.7%). This could be explained by the confounding effects of radiotherapy but needs further analysis.^41^

A meta-analysis of pivotal studies involving osimertinib reported an increased risk of HF (about 19.3%), which developed on an average of 29 days after the initiation of therapy.^42^ The AURA3 Randomized Phase III Study (Osimertinib *vs* Platinum-Pemetrexed) and the FLAURA trial (osimertinib *vs* first-generation EGFR-TKI) in EGFR-Mutated Advanced non-small cell lung cancers (NSCLC) reported the frequency of LVD at 5% and 10%, respectively.^43^ Paradoxically, a larger population study with a 2-year follow-up in 942 gastrointestinal stromal tumor (GIST) patients postulated that imatinib does not significantly induce cardiac failure. Instead, as an add-on therapy for pulmonary arterial hypertension (PAH), it improves exercise capacity, cardiac hemodynamics, right ventricular (RV) function, and left ventricular (LV) early diastolic relaxation.^44^

A combination of BRAF and MEK inhibitors is often used to treat metastatic BRAF-mutated melanoma and is associated with cardiotoxicity in 5% to 11% of patients. However, BRAF monotherapy is rarely associated with LVD.^45^ The COLUMBUS trial conducted in BRAF mutant metastatic melanoma patients investigating a combination of encorafenib plus binimetinib/encorafenib/vemurafenib showed a 6% incidence of all-grade, 6% grades 1–2, and 2% grade 3 LVD.^46^

### Coronary artery disease and myocardial ischemia/infarction

VEGF inhibition is known to increase mitochondrial superoxide generation and decrease nitric oxide (NO) production.^47^ This results in the acceleration of atherosclerosis in apolipoprotein E (apo E) knockout mice with no discernible effects on plaque vulnerability.^26^ Experiments in rats treated with sunitinib demonstrated reduced vasorelaxation due to a reduction in endothelial NO release.^48^ Small molecule TKIs such as ponatinib and nilotinib are associated with increased incidence of myocardial ischemia or infarction.^49^ Ponatinib is also associated with an increased risk of angina pectoris. In addition to acute coronary syndromes, nilotinib has off-target vascular pro-atherogenic properties causing arterial stenosis and vasospasm.^46^ Erlotinib and sorafenib exhibited an increased incidence of cerebrovascular accidents and myocardial infarction (MI).^49^ It is important to note that biologics such as bevacizumab are very target-specific, and their side effects are mostly related to exaggerated pharmacological effects. On the other hand, small-molecule drugs such as sunitinib, sorafenib, and nilotinib are more prone to induce harmful non-target effects.^50^

### QTc prolongation and cardiac arrhythmias

The incidence of QTc prolongation and various arrhythmias with VEGFI is about 0.1%.^51^ Some reports suggested that the QTc interval is unaffected by VEGF inhibition.^26^ No correlation was reported between the duration of drug exposure and QT prolongation effect.^21^ Studies have shown that the effect on QTc interval differs in the same drug depending on indications, duration of therapy, drug combinations, and pathophysiology of the underlying disease.^26^ Infrequently, high-grade atrioventricular (AV) blocks requiring permanent pacemaker implantation have been observed.^52,53^ However, due to the smaller number of studies, the true incidence is yet to be defined. Third-generation EGFR TKIs- rociletinib and osimertinib are reported to have a higher incidence of grade 3–4 QTc prolongation.^43^ Nilotinib has a black-box warning for QTc prolongation and sudden cardiac death (SCD).^54^ Importantly, it has been reported that the QTc prolongation potential is not directly proportional to the increased risk of ventricular arrhythmias.^55^ Concurrent administration of other QTc-prolonging drugs (oral chemotherapeutic agents, antibiotics, psychiatric medications) in cancer patients can have additive effects.^56^

Ibrutinib used in the treatment of chronic lymphocytic leukemia/lymphoma (CLL) is predominantly associated with atrial fibrillation (AF) based on numerous studies.^57,58^ Primarily, AF has been described as an off-target effect of ibrutinib.^59^ In the HELIOS study, AF incidence was higher when ibrutinib was combined with other drugs compared to monotherapy. The median time to onset of AF was around 2.8 months. Ventricular arrhythmias including non-sustained ventricular tachycardia, ventricular fibrillation, and SCD were reported with a cumulative incidence of 1991 events per 100,000 person-years.^60^ An ibrutinib-associated ventricular arrhythmia occurs to a lesser extent compared to AF. No association between ibrutinib and QT prolongation was observed. Paradoxically, there was evidence of QT shortening with ibrutinib use.^55^

Acalabrutinib, a highly selective Bruton’s tyrosine kinase inhibitor (BTKI) was shown to be associated with an increased incidence of AF in the ELEVATE-TN and ASCEND trials (Table 2). The former study included acalabrutinib and obinutuzumab, which were associated with 3% and 4% incidence of AF respectively.^61^ Similarly, the ASCEND trial which compared acalabrutinib with the investigator’s drug of choice demonstrated AF in 5% and 3% of patients, respectively.^62^ The ASPEN study involving a selective TKI, zanubrutinib demonstrated a reduced risk of AF compared to ibrutinib.^63^ However, while considering the results of these studies, we should also acknowledge the pitfalls of these being open-label with no specific screening for AF. Surprisingly, BTKI is known to be associated with a lower incidence of sudden death. A meta-analysis that included 4 studies (RESONATE, RESONATE-2, HELIOS, and RAY) with 1000 ibrutinib-treated patients reported sudden death only in 10 patients.^64^

Bradycardia is mostly associated with ALK inhibitors including crizotinib and ceritinib used in the treatment of NSCLC.^65^ Mechanistically, drug interactions having a significant effect on CYP (Cytochrome P450) 3A4 is reported.^55^ Clinical trials with pazopanib reported sinus bradycardia in about 19% of patients.^26^ However, most patients remained asymptomatic and dose reduction was rarely necessary.^55^

### Systemic arterial hypertension

HTN remains a frequently reported adverse event with TKIs, with evidence suggesting possible dose-independent class effects.^55,66^ A meta-analysis of 77 studies reported a significantly increased risk of HTN, cardiac ischemia, and cardiac dysfunction associated with TKIs.^67^ HTN is usually detected within the first month of treatment. Across different trials, the incidences of HTN with individual drugs were 42% with pazopanib, 63–68% with ponatinib, 7–43% with sorafenib, 5–24% with sunitinib, 40% with axitinib, and 30–59% with regorafenib.^58,68,69^ The highest rate of both all-grade and high-grade (grade 3 or 4) HTN was reported in ibrutinib and lenvatinib. Vandetanib, cabozantinib, and vatalanib were associated with all grades in 24–29% and high-grade HTN in 7–22% of patients, respectively.^66^

Among the small molecule TKIs, VEGFI showed increased association with HTN in about 30–80% of patients.^70^ In a study with metastatic renal cell carcinoma (RCC) patients treated with sunitinib, an average increase in blood pressure (BP) of 14/11 mmHg was observed through 24-h ambulatory monitoring. A new baseline was noted after the first drug cycle, which never returned to the pre-treatment level even after completion of therapy demonstrating a more long-lasting change in vasculature with VEGF inhibition.^55^ Sunitinib was associated with reduced plasma renin concentration and activity with unchanged aldosterone levels suggesting mineralocorticoid-receptor activation.^71^ Alivon et al. noted that sunitinib or sorafenib induces inhibition of vascular signaling pathways increasing arterial stiffness partially independent of the change in BP.^72^ Patients with pre-existing HTN, high body mass index (BMI), advanced age (≥60 years), underlying metastatic RCCs, and use of more potent agents such as axitinib were reported to be more vulnerable.^55,66^

A single-arm cohort study by Dickerson et al. estimated that 78.3% of ibrutinib-treated patients developed a new or worsening HTN (17.7% with BP > 160/100 mmHg) over a median time duration of 30 months. The incidence of HTN is lower in more selective agents compared with ibrutinib.^73^ The cumulative incidence of HTN in ibrutinib was 78% with a median time of onset ranging from 1.8 to 6 months, but it can also develop in a very short time.^60^ ELEVATE-TN and ASCEND trials showed a higher incidence of HTN with acalabrutinib. Zanubrutinib and ibrutinib were studied in the ASPEN trial, where the incidence of HTN was lower in zanubrutinib compared to the ibrutinib group.^57^ Other TKIs including erlotinib, osimertinib, ruxolitinib, and a combination of BRAF and MEK inhibitors showed significant association with systemic HTN.^43,66^

### Pulmonary arterial hypertension

PAH has a relatively lower incidence in TKIs and usually appears as a late complication. Studies have shown a proven risk with dasatinib, and a possible risk with ponatinib, bosutinib, lapatinib, and lorlatinib therapies.^74^ Dasatinib, the second-generation drug used to treat chronic myeloid leukemia (CML) was the first TKI reported to induce severe pre-capillary PAH with an incidence of 0.45% as reported in the French registry.^75^ It usually occurs 8–40 months after the initiation of therapy.^68^ Interestingly, PAH was more frequent in women, and the presence of hormonal or immunological factors may increase the risk.^74^ Mechanistic studies in pre-clinical models demonstrated that dasatinib administered alone was not sufficient for the development of PAH, suggesting the requirement of an additional predisposing factor(s) in the form of pulmonary vascular insult.^74^ In the rat models of pulmonary fibrosis, PAH was exacerbated by VEGF inhibition. In addition to these direct effects, indirect mechanisms such as the development of pulmonary venous thromboembolism in the setting of a cancer-induced hypercoagulable state could be contributory. Studies also suggested that VEGF inhibition can cause the impairment of RV CMs in response to cardiac stress. RV failure can also be explained by the multi-hit hypothesis through CMs, coronary, pulmonary, and venous vasculature modifications.^26^

### Pericardial diseases

Pleural and pericardial effusion is associated with drugs such as dasatinib, nilotinib, imatinib, ponatinib, and FLT3 inhibitors.^58,60^ Dasatinib is frequently associated with pleuropericardial effusion.^76^ FAERS database examining CV toxicities of VEGFI demonstrated a 0.3% incidence of pericardial effusion.^77^

### Thromboembolic diseases

The mechanism of thromboembolism involves the alteration of vascular protective properties of endothelial cells.^66^ Arterial and venous thrombosis results from the reduction of NO synthesis and endothelial dysfunction.^40^ Risk factors include specific cancer types, metastasis, central venous catheter, HF, immobility, AF, obesity, previous episodes of thromboembolic diseases, high-dose chemotherapy, hormonal therapies, advanced age, and the female gender.^58^ The increased incidence of all-grade arterial thromboembolism is predominantly associated with VEGFI including erlotinib, nilotinib, dabrafenib, and trametinib.^28,58^ These agents exacerbate the pro-coagulant state of cancer thus increasing the risk of arterial and venous thromboembolism. Central nervous system (CNS) hemorrhagic or ischemic events are associated with ibrutinib.^78^ Bosutinib used in refractory/relapsed CML is associated with increased cardiac adverse events, especially cerebrovascular events. Ponatinib is reported to have an increased incidence of cerebrovascular (6%) and peripheral arterial occlusive events (8%).^60^

In a meta-analysis including eligible VEGFI patients, an increased risk of MI and arterial thrombotic episodes was observed with therapy but the risk of stroke was not increased.^79^ The incidence of arterial thrombosis is reported at 1.7% for sorafenib and 1.4% for sunitinib.^68^ Ponatinib is associated with venous thromboembolism and is reported in up to 5% of patients. Ibrutinib shows pharmacological interactions with anticoagulants owing to its selective metabolism by CYP3A4, leading to increased plasma concentrations.^60^

To summarize, the main CV complications of cardiotoxic targeted therapies can be classified based on their pathway specificity.^17^ They include-

- *HER2 targeted therapies*: HF, and systemic HTN.^80–82^
- *VEGFIs (also known as angiogenesis inhibitors*: Systemic HTN, LVD and HF, QTc prolongation, and arterial thrombosis including MI.^38,83–85^
- *MKIs targeting BCR-ABL (often called BCR-ABL TKIs)*: Arterial thrombosis leading to MI, stroke and peripheral arterial occlusive disease (ponatinib), venous thromboembolism, systemic HTN, LVD, and HF, accelerated atherosclerosis (ponatinib and nilotinib), QTc prolongation (nilotinib) and PAH (dasatinib).^86–93^

Compared to other TKIs, the VEGFI have wider use in solid tumors of various types due to their broad antiangiogenic activities.^94^ Both VEGFR-antibodies (such as Bevacizumab and Ramucirumab) and small molecule TKIs targeting VEGFR (such as Sunitinib, and Sorafenib) have been used in clinical practice. They however could display different efficacy and toxicity profiles.^95^ While VEGFR-antibodies are very target-specific, and their side effects are mostly related to exaggerated pharmacological effects, small-molecule drugs such as sunitinib, sorafenib, and pazopanib are more prone to induce harmful non-target effects.^50^ In a meta-analysis comparing the efficacy and toxicity profile of antiangiogenic therapies in gastric cancer, VEGR-antibodies were found to be more effective in controlling cancer and showed a better patient survival rate. In addition, a lesser degree of treatment-related adverse events including HTN, proteinuria, and hand-foot syndrome was reported with VEGFR-antibodies.^96^ No differences were noted in terms of bleeding. Antibody development, however, is relatively more laborious and more cost prohibitory compared to the development of small molecule TKIs.

## Pathophysiology and proposed mechanisms of cardiovascular side effects

**Fig. 1:**
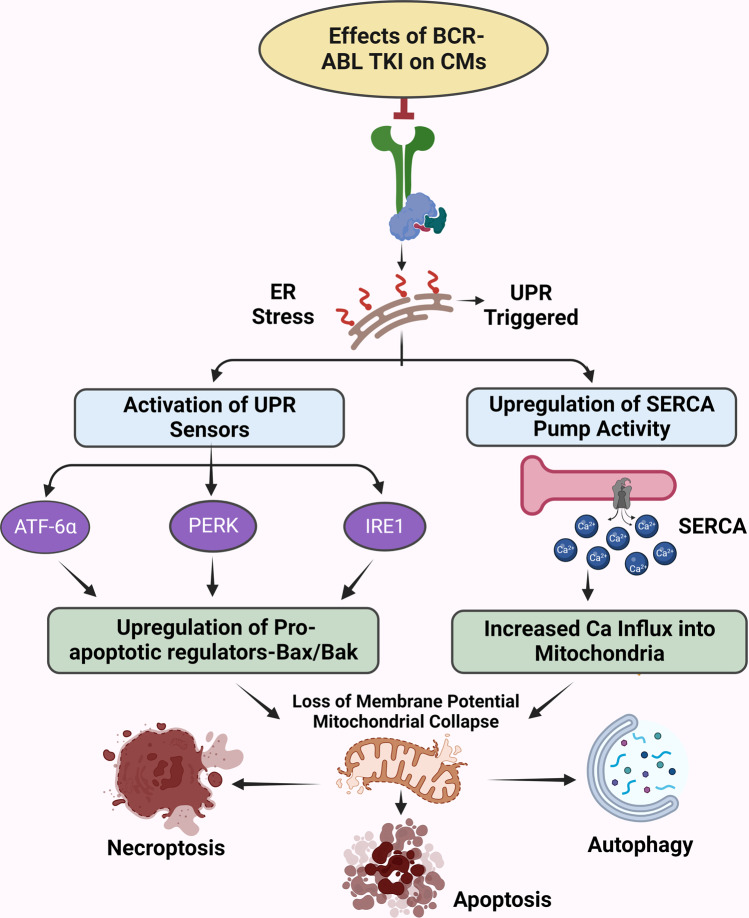
Proposed mechanisms of cardiovascular toxicity induced by *on-target* effects of BCR-ABL TKI. The on-target effect is defined as toxicity arising from the inhibition of the intended tyrosine kinase targets. Inhibition of BCR-ABL TK activity induces prolonged ER stress leading to stimulation of unfolded protein response (UPR) pathways. The UPR pathways constitute 3 major arms, including protein kinase RNA-like endoplasmic reticulum kinase (PERK), inositol-requiring enzyme-1 α (IRE-1), and activating transcription factor 6 α (ATF6-α) transmembrane proteins of ER. When activated, PERK phosphorylates the eIF2α factor, which in turn leads to attenuation of protein synthesis. IRE-1-mediated downstream target activation of JNK signaling promotes both apoptotic and non-apoptotic cell death. ATF6 is a basic leucine zipper transcription factor activated by translocation to the Golgi apparatus. ATF6 and ATF4 lead to the expression of CHOP, a pro-apoptotic transcription factor inducing cell death signaling. By activating pro-apoptotic proteins and inhibiting anti-apoptotic proteins, it directly activates Bax and Bak on the mitochondrial membrane. Altogether CHOP and JNK signaling produce significant mitochondrial dysfunction due to loss of MP, the release of cytochrome c, and ultimately apoptosis and necroptosis. Additionally, there is an abnormal sarco/endoplasmic reticulum Ca^+2^ ATPase pump (SERCA) activity in response to ER stress specifically due to calcium depletion in ER or activation of the UPR pathway. This results in increased calcium influx into mitochondria, the opening of mitochondrial permeability transition pore with a subsequent decline in ATP concentration, and markedly impaired energy generation. ATF4, Activating transcription factor 4; ATF6, Activating transcription factor 6; BAK, Bcl-2 antagonist/killer; Bax, Bcl-2-associated X protein; BCL-2, B-cell lymphoma 2; BCR-ABL, breakpoint cluster region-Abelson; BCL-2, B-cell lymphoma 2; Bid, BH3 interacting domain death agonist; Bim, Bcl-2-interacting mediator of cell death; Ca^2+^, Calcium; CHOP, C/EBP homologous protein; c-kit, Receptor tyrosine kinase; eIF2α, eukaryotic Initiation Factor 2α; ER, Endoplasmic Reticulum; IRE-1, Inositol-requiring transmembrane kinase/endoribonuclease; JNK, c-Jun N-terminal kinase; MLKL, Mixed lineage kinase domain-like protein; MP, Membrane Potential; PERK, protein kinase RNA like ER kinase; RIPK1, receptor-interacting serine/threonine kinase; SERCA, Sarcoendoplasmic Reticulum Calcium ATPase; SR, Sarcoplasmic Reticulum; TK, Tyrosine Kinase; TKI, Tyrosine Kinase Inhibitor; UPR, unfolded protein response. The figures were created using scientific image and illustration software, BioRender (BioRender.com)

Most of the evidence on the mechanisms of TKI function and their side effects has been derived from preclinical studies. In mouse myocardial tissues, increased expression of various proteins and their transcription factors was detected after TKI therapy. These include NRG1 (paracrine factor), which along with ErbB2 activates the mitogenic pathways crucial for the maintenance of CM health and survival.^97^ Studies also reported JunB expression after erlotinib therapy, forkhead box transcription factor (FOXO3), and SRY- Box Transcription Factor 6 (Sox6) expression in sunitinib-treated hearts.^98^ Examining genome‑wide association studies (GWAS) datasets, Li et al. reported single nucleotide polymorphism (SNP) of the various HF-related genes in subjects treated with sunitinib, pazopanib, sorafenib, dasatinib, and nilotinib.^99^ The role of ROS is also recognized as a mechanism of endothelial and vascular smooth muscle dysfunction resulting from TKI use. ROS accumulation can lead to the production of toxic metabolites leading to unpredictable specificities of targeted tumor antigens which can induce unexpected tumor lysis and cytokine release. This can lead to CM contractile dysfunction, apoptosis, and autophagy (Fig. 1).^100,101^ The cardiotoxic effects are also exerted through TKI activities in cardiac fibroblasts, and endothelial and vascular smooth muscle cells, with some studies suggesting the involvement of immune cells and cardiac progenitor cells.^101^ Cardiac pathologies exhibit regulated cell death in the form of apoptosis, necroptosis, mitochondrial-mediated necrosis, pyroptosis, ferroptosis, and autophagic cell death.^23^ TKIs used in both oncologic as well as other nononcologic conditions can lead to molecular changes in the CMs leading to various pathways related to cell death.^102^

### Effects on cardiomyocyte survival signaling

**Fig. 2:**
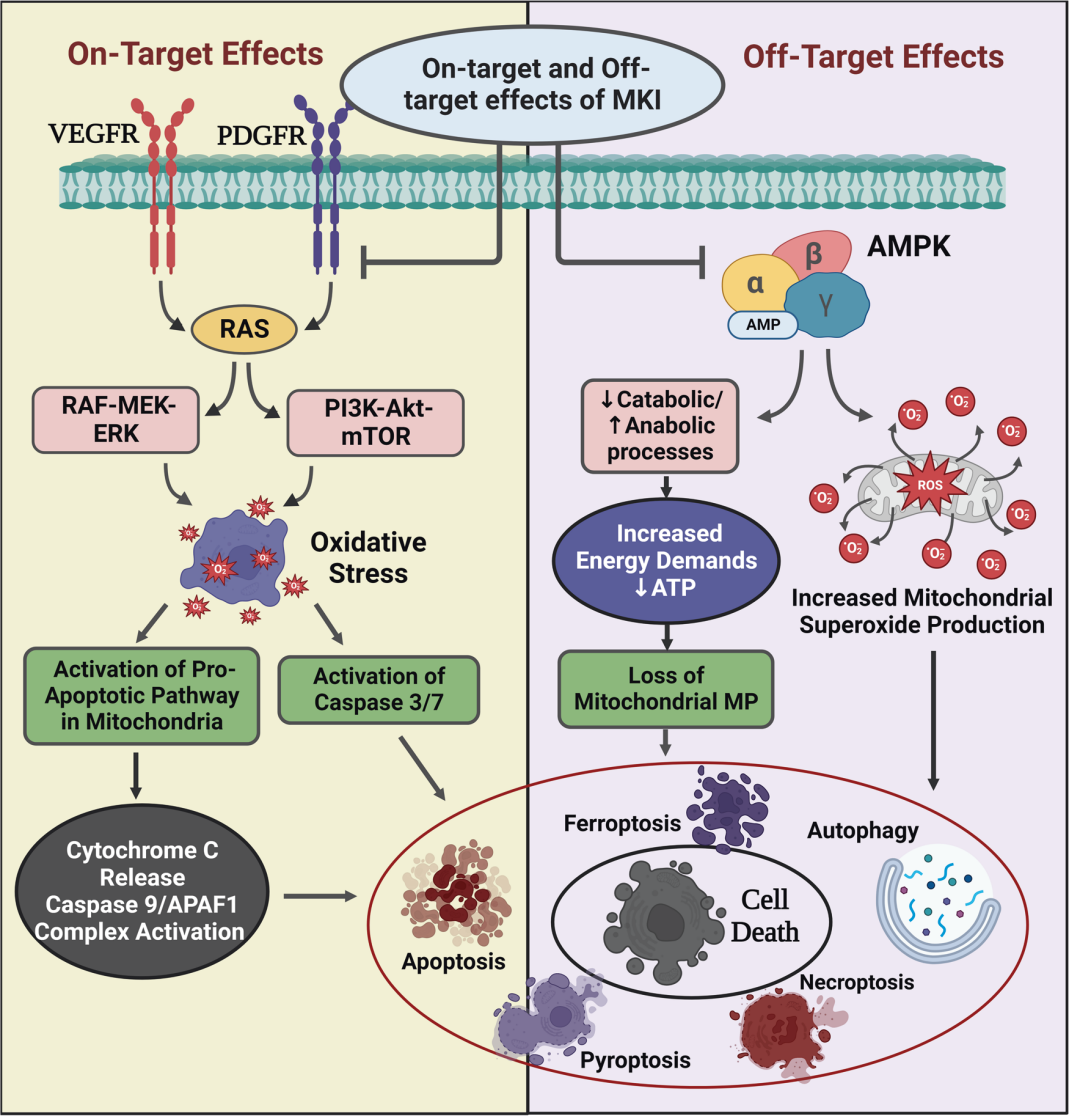
Proposed cellular mechanisms of *on- and off-target* effects of multi-kinase inhibitors. Off-target toxic effects are a result of the inhibition of kinases other than the intended target of the TKIs. Off-target blockage of AMPK by drugs like sunitinib leads to superoxide production in mitochondria resulting in cell death. Additionally, it inhibits catabolic processes such as glycolysis, GLUT 4 expression, and upregulation of anabolic processes such as glycogen synthesis, FA oxidation, and lipolysis which in turn leads to dysregulation of energy homeostasis in CMs along with ATP depletion and loss of membrane potential. It is also known to directly inhibit eIF2, resulting in the impairment of protein synthesis. Its on-target toxicity is mediated through the inhibition of PDGFR and VEGFR, which affects Raf-MEK-ERK and PI3K-Akt-mTOR pathways. This leads to oxidative stress and activation of pro-apoptotic pathways involving the release of Cyt c and activation of caspases and APAF1. Altogether these processes result in increased apoptosis, decreased cell survival and impaired compensatory myocardial response to stress, and ultimately cardiac contractile dysfunction. ATP, Adenosine tri-phosphate; Akt, Ak strain transforming; AMPK, AMP-activated protein kinase; APAF 1, Apoptotic protease activating factor 1; ATP, Adenosine triphosphate; CaMKII, Calcium/Calmodulin Dependent Protein Kinase 2; CM, cardiomyocytes; Cyt C, Cytochrome C; ERK, Extracellular signal-regulated kinase; eIF2, FA, Fatty Acid; GLUT, Glucose transporter; MEK, Mitogen-activated protein kinase kinase; MP, membrane potential; mTOR, mammalian target of rapamycin; P, phosphate; PDGFR, platelet-derived growth factor receptor; PI3K, phosphatidylinositol-3 kinase; Raf, rapidly accelerated fibrosarcoma; Ras, Rat sarcoma virus; ROS, reactive oxygen species; TKI, tyrosine kinase inhibitors; VEGFR, Vascular Endothelial Growth Factor Receptor. The figures were created using scientific image and illustration software, BioRender (BioRender.com)

Dual action of ponatinib induces inhibition of pro-survival pathways (AKT and ERK) and upregulation of pro-apoptotic pathways (Bax, Bcl-xL, and Caspase) was demonstrated by Singh et al. in zebrafish embryos. Importantly, the toxic effects were irreversible even after the drug withdrawal.^103^ These reactions are a result of both on-target and off-target inhibition of signaling pathways (Fig. 2).^104^ Sorafenib, an MKI, concurrently inhibits protein kinase ERK through inhibition of Raf-1 (in response to stressors) and disinhibits pro-apoptotic kinases leading to on-target cardiotoxicity. Dual action involving inhibition of pro-survival signaling pathways and activation of cell death signaling pathways eventually results in apoptosis, autophagy, and necrosis; necroptosis, and ferroptosis. Imatinib suppresses the transcription factor GATA4 by downregulating Bcl-2 and Bcl-xL pathways resulting in cellular apoptosis.^105^

Overexpression of VEGF was shown to be associated with increased capillary density and mitochondrial energetics.^106^ The inhibition of the VEGFR signaling pathway, on contrary, is associated with left ventricular hypertrophy which is a prognostic factor for CV mortality. Animal experiments also demonstrated that VEGF signaling has a significant role in normal cardiac physiology extending beyond angiogenesis.^107^ In clinical practice, CV events are reported to be associated with all approved anti-VEGF agents. Sunitinib and sorafenib can have a direct toxic effect on CMs, mediated either through cell damage or by inhibiting the tissue repair process.^108,109^

### Effects on mitochondrial and sarcoplasmic reticulum homeostasis

TKIs are responsible for mitochondrial oxidative/nitrosative stress through a disproportionate production of ROS/reactive nitrogen species and increased deactivation of antioxidant enzymes, especially in CMs.^40^ This has been exhibited in imatinib and dasatinib by a reduction in membrane potential and complex 1 of the electron transport chain resulting in the cleavage of caspase 3.^110^ Kerkela et al. identified off-target inhibition of AMPK by sunitinib, affecting mitochondrial function important for maintaining CV homeostasis, particularly during increased stress conditions.^111,112^ Sorafenib lowers S16 phospholamban phosphorylation, leading to the reduced sarcoplasmic reticulum (SR) calcium load and delayed removal through SR Ca^2+^-ATPase (SERCA). These effects were reported as rapid, concentration-dependent, and reversible resulting in the net negative inotropy.^113^ Similar mechanisms were also demonstrated with imatinib, dasatinib, and sunitinib therapies^2^ (Fig. 2).

### Effects on cardiomyocyte contraction and excitation

The primary mechanisms of QTc prolongation and cardiac arrhythmias associated with TKIs are postulated to be an *off-target* blockade of the human *Ether-à-go-go*-Related Gene (hERG) encoded potassium (K^+^) channel proteins in CMs which carries the repolarizing rapid delayed rectifier (IKr) current. This results in impaired depolarization and delay in impulse conduction.^39^ An alternate hypothesis suggests that protein misfolding due to specific drug activity or modification of chaperone interactions with other proteins results in the inhibition of sorting of the K^+^ channel proteins. This interferes with the chaperones of hERG channels leaving the ER leading to a reduction of flow through the K^+^ channels.^57^ Adverse effects are also attributed to the downregulation of PI3K/Akt pathways resulting in the regulation of various channel-forming proteins responsible for cardiac arrhythmias. Their direct inhibition activates late sodium current (INa-L), affects L-type calcium current (ICa-L), and modulates calcium cycling. This favors QT prolongation, abnormal automaticity, and early and late after depolarization. Impairment of VEGFR, PDGFR, and c-Src leads to contractile abnormalities and a fall in the heart rate.^60^ The arrhythmogenic effects of BTKI, ibrutinib, can be an on-target (BTK-dependent) or off-target (PI3K/AKT pathway or C-terminal SRC kinase-dependent) effect. However, because of its high specificity to the B lymphocyte receptors, off-target effects are relatively lower.^55,114^ On the other hand, VEGFI toxicity is a *multiple-hit* phenomenon leading to micro-and macro-vascular dysfunction, further potentiated by increased afterload due to HTN. It is often associated with mitochondrial ATP (adenosine triphosphate) depletion, activation of pro-apoptotic kinases, and profound vasoconstriction in some cases.^26^

## Risk factors associated with TKI-related cardiovascular dysfunction

**Fig. 3:**
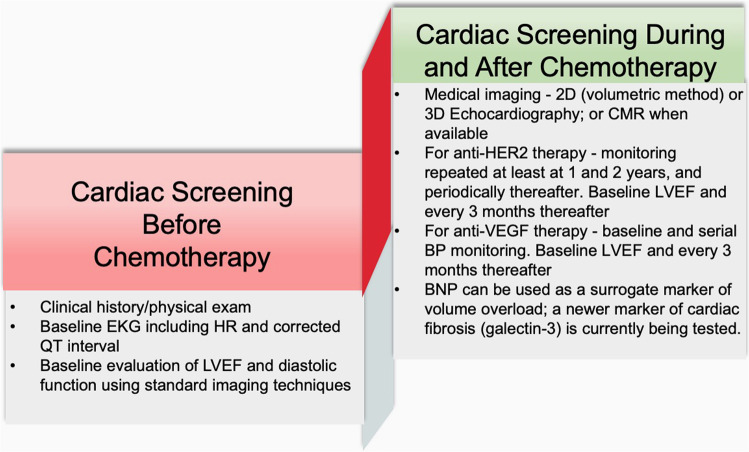
Schematic representation of cardiotoxicity screening before, during, and after cancer therapy. Specific screening criteria based on the types the therapies are proposed. CMR, cardiac MRI; and HER, human epidermal growth factor receptor

*Patient-related risk factors* such as CAD, advanced age, HTN, diabetes mellitus (DM), and smoking.^22^ Other risk factors including atherothrombotic risk can be detected through circulating biomarkers such as high-sensitivity C-reactive protein (hs-CRP), and other markers of inflammation such as interleukin-1 (IL-1), IL-6, and fibrinogen.^115^ Elevated cardiac biomarkers including high-sensitive troponin T (hsTnT), N-terminal pro-BNP (NT-proBNP), mid-regional pro-atrial natriuretic peptide (MR-proANP), mid-regional pro-adrenomedullin (MR-proADM), and C-Terminal pro-endothelin-1 are also considered as the risk predictors.^116,117^ Elevation of these biomarkers in cancer patients, before the initiation of cardiotoxic chemotherapy, is a higher risk of all-cause mortality.^117^ A schematic representation of cardiotoxicity screening before, during, and after cancer therapy is listed in Fig. 3.

*Therapy-related risk factors* include high-dose therapy, administration as a bolus or in combination with other drugs, prior anthracycline use, mediastinal radiation with the heart as a target, and concomitant use of specific agents such as anthracyclines, trastuzumab, and cyclophosphamide are linked to increased risk.^118^ The presence of concomitant HTN before the initiation of therapy is associated with an increased risk of cancer therapy-induced LVD. However, there is no evidence to suggest that the prior HTN treatment reduces risk when compared to other comorbidities such as DM and chronic kidney disease (CKD).^66^ On the contrary, a large retrospective study showed no correlation between TKI-induced cardiotoxicity and CV risk factors suggesting a possibility of genetic predisposition to TRCD in some cases.^119^ CV diseases, chemotherapy-induced cardiotoxicity, and cancer are more common in post-menopausal women (associated with metabolic changes, oxidative stress, and subclinical inflammation). Herrmann et al. proposed an objective Cardiotoxicity Risk score (CRS) which estimates the risk by considering both patient and therapy-related risk factors. Considering such risk predictive algorithms, ponatinib, sunitinib, and sorafenib are *moderate-risk;* whereas imatinib, lapatinib, and dasatinib are *low-risk*.^22^

## Schema for the clinical management of TRCD

Early detection and prompt therapy for cardiotoxicity provide opportunities to prevent irreversible cardiac damage. Early cardiotoxicity is often asymptomatic or subclinical with no structural damage present and can be reversible. The late-stage cardiomyopathy results from irreversible structural changes and patients are often symptomatic.^100^ The most recent American and European guidelines (American Society of Clinical Oncology-ASCO-2017 and ASCO-2018), European Society for Medical Oncology (ESMO-2017, ESMO-2020), and European Society of Cardiology (ESC-2016) were analyzed by a cardio-oncology expert panel from the French Working Group of Cardio-Oncology. They have put forth *cardio-oncological evaluation protocols* for the proper screening and management of patients exposed to cardiotoxic agents.^28^

### Pre-treatment assessment and prevention

The key principles include screening and management of pre-existing CV risk factors (Age > 60 years, CAD or MI, AF, HF, tobacco use, hyperlipidemia, HTN, DM, and obesity).^28,55,68^ Screening before anticancer therapy including baseline risk assessment is imperative in all patients before the initiation of therapy, focusing on early pre-clinical detection of cardiotoxicity.^119^ This may significantly reduce the possibility of their occurrence during treatment and helps in identifying patients who would benefit from cardioprotective therapy and adjustment of therapy before irreversible injury occurs.^28,119^ BP measurement, baseline electrocardiogram (ECG), serum fasting lipid profile, HbA1C, glomerular filtration rate (GFR) calculation, and cardiac biomarkers should be considered.^28,55,120^

The HF status and its underlying causes (including CAD and valvular heart disease) should be evaluated as part of baseline screening. In the case of CAD, provocative testing is recommended to rule out any residual disease.^121^ The severity of valvular diseases should be assessed to determine the requirement of correction before the initiation of cancer therapy. In the case of heart failure with reduced ejection fraction (HFrEF) and left bundle branch block (LBBB), cardiac resynchronization therapy (CRT) should be considered.^122^ The novel CV risk stratification model which was proposed by the Cardio-Oncology Study Group of the Heart Failure Association (HFA) and ESC in collaboration with the International Cardio‐Oncology Society (ICOS) was used to stratify the CV risk. In studies with CML patients, the HFA-ICOS baseline stratification proforma has proven to be more sensitive than the older Systematic Coronary Risk Evaluation (SCORE) charts.^123^

### Cardiac functional assessment

In patients treated with targeted therapies, a sustained low normal left ventricular ejection fraction (LVEF) is considered to be of high risk.^45,119^ LVEF of 53% was considered a key metric to discern normal function *vs*. cardiac dysfunction, as proposed by the American Society of Echocardiography (ASE) and the European Association of Cardiovascular Imaging (EACVI).^124^ Global longitudinal strain (GLS), a parameter of myocardial deformation, is also used as an early cardiac imaging parameter to assess TRCD. A GLS below 18% or more than 15% drop from the lower limit of normal (LLN) is considered abnormal.^27^ Recommendations from the ESC, ASE, EACVI, and ESMO state that a reduction of LVEF by more than 10% from baseline to a value below the LLN, or more than 15% relative reduction in GLS from the baseline suggests LVD.^125^ Tissue Doppler imaging (TDI) is also used to evaluate diastolic dysfunction with indexes such as septal and lateral-mitral annular e’ velocity and average E/e’ ratio used for prognosis.^126^

The general principles of cardiac imaging related to cardiotoxicity include BP measurement during echocardiography (ECHO), a combination of clinical and biomarkers with imaging, measurement of 2-dimensional (2D) (modified biplane Simpson’s technique) or three-dimensional (3D) LVEF, LV volumes and GLS and cardiovascular magnetic resonance (CMR) in some instances.^27,55^ Currently, techniques such as 3D ECHO and 3D speckle tracking ECHO are used depending on the prior CV history and planned therapies with single or combined cardiotoxic agents.^100^ CMR imaging is an emerging advanced cardiac imaging tool considered a gold standard to assess changes in ventricular volumes and EF is suggested in cases of substandard ECHO images if a discrepancy between LV function measurements exists or in patients with pre-existing complex heart diseases or if ischemic myocardial perfusion assessment is simultaneously planned.^27,55^ It provides better spatial resolution compared to 2D which is highly accurate and reproducible.^55^ It also offers helpful information regarding the presence of prior MI scar, diffuse fibrosis, and intercellular or interstitial edema during cancer treatment. ASE/EACVI guidelines require CMR if LVEF is close to 53% or the ECHO image is of poor quality.^127^

### Role of biomarkers

Cardiac troponin I and brain natriuretic peptide (BNP) can be used to detect myocardial injury and elevation in LV filling pressure and wall stress, respectively.^119,128^ While no specific biomarkers are identified with TRCD, a rise in troponins is often reported. A rise in troponin occurs early, within 12 hours in 53% of patients and a rise within ≈3 days of high-dose chemotherapeutic drugs is highly predictive of cardiotoxicity.^55,119^ Therefore, it is important to have the baseline biomarkers profile followed by sequential measurements at the appropriate intervals. According to ESC and American College of Cardiology (ACC) guidelines, the measurement of B-type NP (BNP) and N-terminal pro-BNP (NT-proBNP)^28,68^ is an HF criterion and plays an important role in monitoring for chemotherapy-related HF.^129^ Novel Biomarkers such as C-reactive protein (CRP), IL-6, myeloperoxidase (MPO) for inflammation; Plasminogen activator inhibitor (PAI), tissue-type plasminogen activators (tPA), and soluble intercellular adhesion molecule (ICAM) for endothelial dysfunction, glycogen phosphorylase BB, and NRG-1 for myocardial ischemia; circulating microRNAs, galectin-3 for myocardial remodeling and development of cardiac fibrosis; and sST2 for cardiac remodeling have also been reported.^130–132^

To conclude, the decision to treat patients with TKI therapy is based on a multitude of factors including the urgency of cancer care, the availability of alternative agents, and the level of risks. HFA of the ESC Cardio-Oncology Study Group in collaboration with the ICOS had developed a baseline CV risk assessment proformas for seven cardiotoxic cancer therapy classes known to cause a range of CV toxicities.^28^ Separate proformas are used for these seven drug classes including *Anthracycline chemotherapy, HER2 targeted therapies, VEGFIs, MKIs for CML targeting BCR-ABL, MKIs for CML targeting BCR-ABL, Proteasome inhibitors (PIs) and immunomodulatory drugs (IMIDs), Combination RAF and MEK inhibitor treatment, Androgen deprivation therapies (ADT), Immune checkpoint inhibitors*. Completion of the baseline CV risk assessment proformas in all patients scheduled to receive one of the seven oncology drug classes with potential cardiotoxicity is recommended. *Low-risk* level cancer patients continue with treatment with CV surveillance as appropriate according to local, national, and international guidelines. *Medium-risk* cancer patients require closer monitoring of CV health during treatment or consideration for referral for a cardio-oncology or cardiology assessment. *High* and *very high-risk* level patients are referred for a cardio-oncology or cardiology assessment, ideally in a specialist cardio-oncology service (if available) to optimize the management of their pre-existing CV disease and modifiable CV risk factors and provide a personalized management plan for surveillance during cancer treatment.^17^

## Primary prevention in high-risk patients

Strategies to reduce cardiotoxicity risk include encouraging a healthy lifestyle and identification and management of risk factors.^119^ Secondary causes of HTN, including untreated obstructive sleep apnea (OSA) should be considered.^55^ Other considerations include limiting or using lower doses of anti-cancer drugs when feasible. Cardioprotective drugs include dexrazoxane, beta-blockers (BB) (prevent LVEF reduction and decrease the incidence of HF), angiotensin-converting enzyme inhibitor (ACEI) (prevent LVEF deterioration), and combination therapies (shows no reduction in LVEF in 6 months).^119^ BP of below 140/90 mmHg is advocated for all patients; 130/80 mmHg in case of CKD or DM.^32^ Chang et al. suggested that cardio-protection should be considered in patients with EF < 50% or a drop of > 10%, GLS > 15% drop, and elevated cardiac biomarkers.^49^ All patients exposed to cardiotoxins should be treated as American Heart Association (AHA) HF stage A-at risk for HF but without structural heart disease.^22^ ACEI/angiotensin receptor blockers (ARBs) and BB (newer generation such as carvedilol and nebivolol) combination therapy are recommended in cancer patients with an LVEF of <40% and also in patients with an asymptomatic decline in LVEF ( < 10% decrease from baseline or <53%) by the Canadian Cardiovascular Society.^40,128^

Baseline BP should always be measured before the initiation of TKI therapy, and every week thereafter for the first 8 weeks and before any infusion or treatment cycle.^133^ In the presence of cardiac risk factors (pre-existing CV disease, CKD, DM, age ≥ 75, Systolic BP ≥ 130 mmHg), antihypertensive medications should be started before chemotherapy.^32,57^ The BP ideally should be maintained below 140/90 mmHg but in the case of CKD, it should be <135/85 mmHg.^133^ Both HTN and ibrutinib are independent risk factors of AF, therefore the ideal BP to initiate antihypertensives is ≥140/90 mmHg.^57^ Spironolactone, a mineralocorticoid antagonist with the potential to reduce fibrosis in HF can also play a role in cardioprotection.^128^ Rao et al. recommended a baseline fasting lipid profile and prophylactic statin and aspirin therapy in BCR-ABL TKIs which have an increased risk of atherosclerosis.^55^

In patients with drug-induced AF, thromboembolic events can be controlled by using LMWH (Low Molecular Weight Heparin) for the short-term, and warfarin and DOACs (Direct Oral Anticoagulants) can be utilized for long-term therapy.^120^ HF has an increased incidence of venous thromboembolism. DOACs are beneficial in these conditions showing efficacy, and safety compared to warfarin. Since the long-term safety of DOACs is still under investigation, LMWH remains the preferred option for prophylaxis.^122^ A few cancer types are associated with bleeding themselves and the agent used should be weighed against the risk associated, and the presence of drug-drug interactions carefully evaluated.^120^

## Cardiac surveillance in patients receiving TKIs

There is no standardized protocol currently available regarding surveillance.^45^ In the case of VEGFI and BCR-ABL TKI-treated patients, an ECHO should be repeated at least every 4 months for the first year. However, in high-risk patients, an early assessment is done 2 to 4 weeks after the initiation of drug therapy. In patients undergoing long-term therapy who remain asymptomatic during the first year, an ECHO can be done every 6–12 months thereafter.^27,120^ BCR-ABL TKIs are primarily metabolized by CYP3A4 and their levels are increased when administered concomitantly with inhibitors of CYP3A4 such as diltiazem and verapamil.^134^ Peripheral atherosclerosis is increasingly associated with BCR-ABL TKIs such as nilotinib and ponatinib.^135^ Ankle Brachial Index (ABI) is highly sensitive and specific and a value of <0.9 is specific for peripheral arterial disease.^136^ ABI (or duplex ultrasonography) is assessed every 6–12 months as per the European LeukemiaNet recommendations.^137^

The drugs used for the chemotherapy regimen should be selected based on their known effects on pre-existing CV disease. If a cardiotoxic drug is required, effective management of the pre-existing comorbidities is essential.^115^ For example, the interrupted HER2 targeted can be resumed, once the LVEF improves.^28^ However, it is usually avoided when LVEF is less than 40% unless no effective alternative treatment exists.^120^ It is important to note that the toxicity due to HER2 TKI therapy is often irreversible.^114^ During therapy, asymptomatic low-risk patients require no follow-up, however medium- to high-risk patients require CV assessment after the last dose, at 3–6 months, and then at 12 months. In patients with new symptoms of LVD, the safety of continuing therapy should be evaluated. During the long-term therapy in asymptomatic metastatic diseases, a 3-month follow-up during the first year is recommended. Thereafter, if the cardiac functional parameters are within the normal range (low risk) less frequent follow-up is recommended.^27^

For cardiac surveillance, the screening test of choice is a transthoracic echocardiogram (TTE). Other modalities include ventilation-perfusion (VQ) scan to study pulmonary embolism and right heart catheterization (RHC) to establish the presence of PAH (increase in the mean pulmonary artery pressure ≥ 25 mmHg at rest and pulmonary capillary wedge pressure < 15 mmHg).^58,115^ RV function and estimation of pulmonary pressure using ECHO with spectral Doppler screening are required to establish the pre-treatment baseline, detect pre-existing PAH, and if cardiac symptoms develop, maintain a low threshold for repeat ECHO. EACVI suggests that routine RV-free wall strain measurement is more characteristic of RV longitudinal deformation than septal strain. Currently, the estimation of RV ejection fraction is possible by both CMR and 3D ECHO.^138,139^ Routine monitoring for right HF and PAH is recommended only in patients with cardiopulmonary symptoms before the initiation of therapy, and every 3 months thereafter as per ESC.^115^ TKI-induced PAH can be fatal and therefore early screening and prompt management are required.^74^

The German consensus guideline recommends ECHO after a month of starting BRAF and MEK inhibitors, and thereafter every 3 months during therapy. Treatment can be withdrawn if LVEF is below 40% or when there is an incremental decrease of >10% in the setting of LVEF of 40–49%.^45^ In ibrutinib-treated patients, in addition to the baseline evaluation, they require further monitoring every 3 months in the first year. This is advantageous since most of the rhythm disorders associated with the drug therapy occur with the peak incidence within the first 30 days. AF, HF, and ventricular arrhythmias are diagnosed within the first 2–3 months. Additionally, drug-induced HTN is established within the first 4–5 months of therapy, and most of the abnormal echocardiographic findings are noted during the first year.^140^ If patients are symptomatic, Holter monitoring along with 12-lead ECG is recommended.^120^ Diagnosis of deep vein thrombosis (DVT) is made by compression ultrasound and pulmonary embolism is by spiral CT (computed tomography) angiography (CTA). VQ scan has a low sensitivity and specificity. When allergic to iodinated contrast, magnetic resonance (MR) pulmonary angiography is employed.^58^ D-dimer levels are associated with poor prognosis in the setting of cancer, independent of venous thromboembolism. They can be used to rule out venous thromboembolism, but not for diagnosis. The proposed optimum cut-off is between 981–1,500 ng/mL but these values are not externally validated.^129,141^

## Management of TKI-induced hypertension and cardiac dysfunction

Regular monitoring for HF during chemotherapy helps early detection of cardiotoxicity either leading to the continuation of cardioprotective measures or permanently discontinuing chemotherapy.^122^ The presence of HF is not always a plausible reason for the interruption of chemotherapy.^120^ The benefit versus the potential ill effects of the continuation of therapy should always be considered.^55^ Cancer drugs can be continued in the absence of a hypertensive emergency or HTN-mediated end-organ damage along with prompt initiation of HTN management and optimization.^142^ The goal is to maintain BP < 140/90 mmHg in uncomplicated HTN and <140/85 mmHg in patients with DM and renal failure.^119^ Most of these patients can be managed by dose modifications and normal anti-hypertensive medications (ACEI, ARBs, BBs, amlodipine, and aldosterone antagonists).^119,133^ Some studies suggested that the presence of HTN serves as a biomarker for treatment efficacy.^28,133^ Renal findings including proteinuria >1 g/d, hematuria, or acute renal failure will require a nephrology referral. Cancer treatment can be safely resumed once the BP management is within the expected goal.^120^

ACEI and BB are the first-line treatment in HF or LVD with HTN, especially with VEGFI.^68,120^ Non-dihydropyridine calcium channel blockers (DHP CCBs) are avoided owing to drug-drug interactions.^120^ It is suggested that greater efficacy and lower adverse effects are seen with lower-dose antihypertensive combination therapy.^55^ HTN in the setting of VEGFI can be a sign of its efficacy and should not dictate mandatory discontinuation.^143^ It should be interrupted and evaluated to see if resumption is appropriate.^28^ Treatment of HTN has significant beneficial effects on major adverse cardiovascular events (MACE), such as CAD, HF, stroke, end-stage renal failure, and overall mortality.^66^ HTN can develop from the initiation of therapy until one year after therapy, especially in sunitinib.^68^ TKI therapy can be continued as long as there is no severe HTN. In case of proteinuria, ACEI, ARBs, or DHP CCBs can be given.^133^ Non-DHP CCBs are contraindicated with oral angiogenesis inhibitors since these agents inhibit CYP3A4 resulting in increased VEGFI levels.^133^

### Management of arrhythmias

Caution is advised when combining antiarrhythmic agents with cancer therapy owing to drug-drug interactions and QTc prolongation.^120^ ECG is recommended at baseline, 7 days after initiation, after every treatment cycle, after initiation of new medication, when plasma concentration reaches a steady state, after any dose adjustments, or in the event of electrolyte imbalance.^137,144^ In patients with QTc prolongation, correctable causes like electrolyte abnormalities (hypokalemia, hypomagnesemia, hypocalcemia) should be identified and corrected (Magnesium > 2.0 mEq/l, Potassium at > 4.0 mmol/l) before starting chemotherapy.^55,144^ In general, cessation of cancer therapy due to any cardiotoxicity should be considered only if all other alternatives have been exhausted.^49^ BTKIs, apart from AF, possess an antiplatelet effect leading to increased bleeding risk; therefore, caution should be exercised when combining ibrutinib with antithrombotic agents.^145^ The majority of cases can be managed without the interruption of therapy. Alternatives include more selective agents (acalabrutinib, zanubrutinib) and venetoclax-based strategies.^57^

### Management of pleural and pericardial effusion

The reduction of risk of pleural and pericardial effusion can be achieved by dose reduction of drugs such as nilotinib, ponatinib, or FLT3 inhibitors or using a single dose of dasatinib in a day.^60^ When effusion occurs, apart from discontinuing dasatinib, diuretics or a short course of steroids can be helpful for reversal.^146^ Emergency pericardiocentesis is required in response to a large pericardial effusion (≥2 cm) of hemodynamic significance or less emergent for the diagnosis. Surgery is required to create a pericardial window for recurrent pericarditis.^58^

### Management of pulmonary arterial hypertension

In the case of PAH, permanent discontinuation of dasatinib is necessary.^146^

### Management of thromboembolism

The previous history of arterial thromboembolism is not an absolute contraindication for VEGFI but if an event occurred within the last 6–12 months, caution is warranted. Discontinuation of therapy is only recommended in grade 3 or higher thromboembolic events. Therapy can be restarted once the symptoms resolve.^147^ Antiplatelet and anticoagulant drugs can be given with VEGFI despite the increased bleeding risk. Other drugs include aspirin (prophylaxis in high-risk patients), LMWH, or unfractionated heparin (UFH). When venous thromboembolism develops on chemotherapy, the therapy should be stopped and anticoagulants started immediately. Thrombolytic therapy can be added if necessary. Cancer therapy is restarted once stabilization and anticoagulation are achieved.^58^

## Post-treatment management

Cancer survivors require long-term care for therapy-induced CV comorbidities. Management of LVD is carried out in line with AHA/ACC HF guidelines and individualized according to the patient’s risk factors.^148^ In a large prospective cohort study involving non-metastatic breast cancer patients, a strong inverse relationship was reported between exercise intensity and CV conditions such as CAD and HF.^128^ A routine exercise with a goal of ≥ 9 MET-hour per week showed a reduction in CV morbidities in a prospective study involving 2,973 non-metastatic breast cancer patients. Exercise has been recommended for patients in cardiac rehabilitation with an increased risk of cardiotoxicity by AHA and all survivors of cancer by ESMO.^149^

**Fig. 4:**
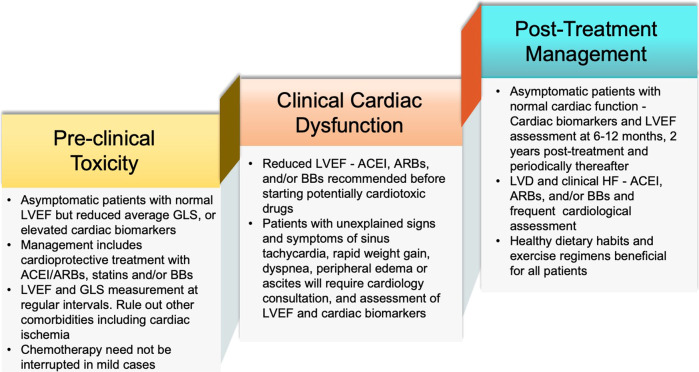
Management of cardiotoxicity based on the clinical manifestations and the severity of the cardiotoxicity. It is recommended to utilize identical imaging modalities, biomarkers, and other laboratory parameters during different follow-up stages for comparability. Reduction in GLS is considered significant when there is a ≥ 12% relative decrease or **≥** 5% absolute decrease. 2D- 2-dimensional; 3D, 3-dimensional; ACEI, Angiotensin Converting Enzyme Inhibitor; ARB, Angiotensin Receptor Blockers; BB, Beta Blockers; CMR, Cardiac Magnetic Resonance imaging; GLS, Global Longitudinal Strain; HER, human epidermal growth factor receptor**;** IHD, Ischemic Heart Disease; PAD, Peripheral Artery Disease; and VEGF, Vascular Endothelial Growth Factor

Long-term surveillance is recommended for patients with pre-existing conditions and also to assess the risk for late cardiotoxicity.^150,151^ In HER2-targeted therapy, assessment should be carried out right after treatment and at a 3-month follow-up. CV evaluation including detailed clinical history and physical examination should be undertaken.^152^ When cardiotoxicity is suspected, further testing in the form of imaging [ECHO or cardiac magnetic resonance imaging (MRI)] is recommended. If asymptomatic, further testing is unnecessary.^128^ An outline for the management of cardiotoxicity is depicted in Fig. 4.

## Gastrointestinal adverse reactions

Gastrointestinal (GI) symptoms are frequently reported in cancer patients treated with TKIs.^153,154^ Mild diarrhea with a grade of 1–2 CTCAE (Common Terminology Criteria for Adverse Events) and colitis were identified as the most common occurrences.^155^ Other symptoms include nausea, vomiting, stomatitis, mucositis, dysgeusia, dyspepsia, anorexia, constipation, abdominal discomfort, and weight loss (Table 1).^156^ These symptoms were considered non-specific due to their common occurrence in most of the chemotherapeutic drugs utilized in the treatment of solid organ, hematologic and endocrine malignancies.^155^ The majority of these side effects were rapid-onset, mild, and often self-limiting. They persisted for a short duration and were mostly TKI dose-related.^157^ Importantly, high rates of incidence and severity, but reduced rates of recurrence of GI side-effects were noted in solid organ cancers such as genitourinary and lung compared to hematologic malignancies.^158^ This explains the rationale behind frequent dose reductions of TKIs in solid organ cancers with better tolerance in hematologic cancers. Other more severe manifestations such as colonic perforation and severe and life-threatening diarrhea and colitis were reported in some cases.^159–162^

### Mechanisms of GI side effects

**Fig. 5:**
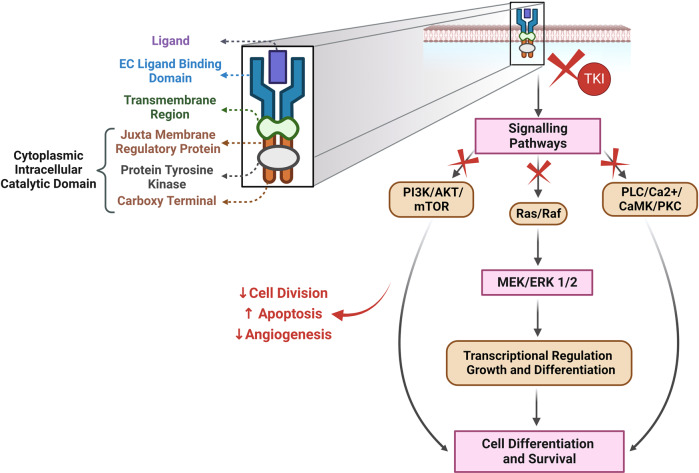
Receptor tyrosine kinase and its downstream pathways affected by tyrosine kinase inhibitors: Upon binding with the ligand the tyrosine kinase receptor gets activated resulting in the activation of multiple pathways, which are critical in cell survival, growth, and differentiation. Aberrant activation of these tyrosine kinase pathways is implicated in carcinogenesis. These effects are blunted by tyrosine kinase inhibitors (TKIs), which not only affect tumor growth but also hinder the function of normal cells in various organs, including cardiomyocytes in the heart. EC, extracellular; Akt, Ak strain transforming /protein kinase B; RAF, rapidly accelerate fibrosarcoma; PI3K, phosphoinositide 3-kinase; mTOR, mammalian target of rapamycin; Ca^2+^, Calcium; CAMK, calmodulin-dependent protein kinase; CM, cardiomyocyte; EGFR, epidermal growth factor receptor; ERK, extracellular-signal-regulated kinase; MEK, Mitogen-activated protein kinase kinase; PKC, Protein kinase C. The figures were created using scientific image and illustration software, BioRender (BioRender.com)

The potential mechanisms are widely debated, though many theories have been proposed. Diarrhea may be a consequence of EGFR-related inhibition of epithelial growth and limited healing of the GI mucosa lining.^163^ Other mechanisms including direct toxic effects on mucosal cells (Fig. 5) and increased GI inflammation have also been implicated.^164,165^ Endoscopic luminal appearance and inflammatory findings on histology are attributed to the direct toxicity but these mechanisms are yet to be confirmed.^166^

BCR-ABL TKIs and BTKIs exhibit GI toxicity during the initial months of therapy, or after any change in the therapeutic algorithm.^167,168^ Stomatitis is frequently associated with fibroblast growth factor receptor (FGFR) TKIs with the inflammatory lesions reported as well-defined and painful.^169^ About 12% of patients treated with regorafenib to 58% treated with erdafitinib reported stomatitis which was self-limiting but painful, affecting the quality of life.^170^ Bosutinib is reportedly associated with diarrhea.^171^ The ENESTnd and DASISION trials in CML patients reported a higher GI toxicity profile associated with imatinib compared to nilotinib and dasatinib.^172^ Ponatinib, used after treatment failure with imatinib, was associated with abdominal pain and constipation.^173^ As part of phase III clinical trial, all-grade diarrhea was increasingly associated with second-generation irreversible EGFR TKIs compared to reversible inhibitors.^174^ In studies involving VEGFI, sorafenib, regorafenib, lenvatinib, and cabozantinib, rates of nausea and vomiting were higher.^175^ Furthermore, drug combinations such as BRAF and MEK inhibitors reported more frequent episodes of nausea, vomiting, and diarrhea than alone.^176^

### Clinical management

Metoclopramide, levosulpiride, or ondansetron can be used as antiemetics. However, caution should be exercised in the case of ondansetron given its interactions with TKIs and the additive effect on QTc prolongation.^175^ Prophylaxis of stomatitis/mucositis involves maintaining good oral hygiene, non-alcoholic mouthwashes, and consumption of non-irritating food to avert stomatitis. Guidelines for fungal, viral, and/or bacterial prophylaxis with topical or systemic antimicrobials. Grade 2/3 stomatitis warrants dose reduction or drug cessation with no interventions needed for grade 1 stomatitis. Diarrhea can be managed with dietary modifications including a low-fat, low-fiber diet. Any underlying infection or other causes of diarrhea should be further examined.^175^ Fluid status should be monitored and adequate hydration maintained.^177^ Diarrhea usually resolves with supportive treatment, and immunosuppressive medications or antibiotics are usually not needed.^178^ Severe or persistent episodes, though uncommon, necessitate medication switches or discontinuation.^173^ In patients treated with erlotinib and afatinib, dose reductions hasten treatment, but not with gefitinib.^100^ Severe manifestations, such as GI perforation, warrant immediate drug cessation.^154^

## Hepatotoxicity

TKIs are associated with frequent elevations of hepatic enzymes, mainly aspartate aminotransferase (AST), alanine aminotransferase (ALT), and gamma-glutamyl transferase (GGT) during the initial months of treatment.^179^ The highest levels of elevated hepatic enzymes were reported in nilotinib, bosutinib, and ponatinib.^180^ Nilotinib is also known to cause bilirubin elevations. In general, dose reduction is not necessary since modest hyperbilirubinemia does not constitute toxicity (Table 1).^181^ Imatinib and ponatinib are associated with late-onset hepatotoxicity and rare cases of hepatic failure.^182^ Severe cases have been reported when imatinib is concurrently taken with acetaminophen.^173^ Studies with EGFR TKIs, particularly gefitinib, and erlotinib can cause transient elevations of enzyme levels without clinical manifestations. Toxicity is more prominent with specific EGFR mutations in patients of Asian descent.^166^ Among ALK TKIs, various clinical trials provided evidence of hepatotoxicity with crizotinib and alectinib, with the higher grades seen in ceritinib and brigatinib (Table 1).^183^ Similarly, ibrutinib and acalabrutinib were more toxic than zanubrutinib, necessitating dose reductions or avoidance.^184^ The association of pazopanib with polymorphism of the UGT1A1 gene in patients with Gilbert’s syndrome was reported by Xu *et. al*. The resultant hyperbilirubinemia was reported as a benign manifestation requiring no interventions.^185^ Additionally, pazopanib is highly protein bound and requires dose adjustment in hypoalbuminemia.^186^

Most cases of TKI-induced liver dysfunctions are asymptomatic and require no treatment.^166^ Baseline liver functions need to be evaluated before the initiation of therapy. During treatment, regular monitoring of hepatic enzymes is needed- biweekly in the first 2 months and every 1–2 months thereafter.^187^ Any major changes (e.g. grade 3 CTCAE) in the levels of the enzymes require dose modifications with reconstitution of regular doses once the values return to grade 1 or baseline levels.^166^ Severe cases of TKI-induced hepatitis require drug substitution.^173^ Caution should be exerted in cases of long treatment duration and concurrent use of other hepatotoxic medications.^166^

## Pancreatic Dysfunction

Acute pancreatitis was observed to a lower extent in TKI-treated patients (ranging from 0 to 4.3%). Increased levels of pancreatic enzymes including serum amylase, and/or lipase levels are reported, which are mostly asymptomatic.^188^ TKIs exert dose-dependent pancreatic toxicity mostly during the initial months of therapy.^173^ The enzyme levels were highest with sorafenib and lowest with nilotinib.^188^ However, in the ENESTnd trial, both imatinib and nilotinib were identified to have very high incidence.^87^

The mechanisms of pancreatic enzyme elevation after sorafenib therapy are unclear. VEGF inhibition results in pancreatic tissue ischemia and acute pancreatitis may ensue.^189,190^ In the case of nilotinib, its high-affinity inhibition of non-receptor TK c-Abl proteins can affect multiple signaling pathways regulating pancreatic cell death. Mechanisms affecting the calcium release and increased accumulation of fatty acid inside the pancreatic acinar cells have also been proposed. It has been documented that monitoring pancreatic enzymes is important during TKI therapy, especially with sorafenib and ponatinib.^191^ Immediate drug cessation is required for grade 3–4 enzyme elevations. On resolution, treatment can be restarted albeit at a lower dose. Switching the drug is necessary for recurrent pancreatitis.^173^

## Dermatological adverse reactions

TKIs are known to be associated with a wide range of mild to moderate skin reactions in more than 70% of patients (with severe reactions in 2–16% of patients) localized to the head and trunk.^166^ Side effects include rash, dry skin, pruritus, and inflammation of nail/periungual tissues as seen specifically in EGFR TKIs and BCR-ABL TKIs.^15,100^ Common cutaneous reactions to EGFR TKIs are known as the PRIDE syndrome (papulopustular and/ or paronychia, regulatory abnormalities of hair growth, itching, and dryness due to the EGFR TKIs).^192^ Toxicity is dose-related and for the most part, self-limited.^173^ The highest incidence of skin lesions is seen in sorafenib and the lowest in sunitinib and vandetanib.^193^ Perifollicular, hyperkeratotic, and erythematous maculopapular eruptions are the most frequent lesions with nilotinib. Skin rashes were frequently reported with imatinib, bosutinib, ponatinib, and cabozantinib (Table 4).^194^ General management involves supportive care with emollients and antihistamines with or without steroids. Severe cases require a change of drug dosage or alternative TKIs.^173^ Treatment regimen with TKIs should be continued in cases of grade 1 and 2 adverse events and discontinued for grade 3 adverse events. When the reactions improve to grade ≤ 1, a reduced dose is again recommended.^170^

**Table 4:** Various Dermatological Lesions Associated with TKIs

Tyrosine Kinase Inhibitors	Associated dermatological lesions
Imatinib	**Maculopapular eruptions** along with pigmentary changes, Photosensitization, Superficial edema affecting the periorbital area (epiphora, chemosis, and conjunctivochalasis), Urticarial, Lichenoid, and Psoriasiform eruptions, Exacerbations of the existing Psoriasis, Hypo-/Hyperpigmentation, Neutrophilic eccrine hidradenitis, and neutrophilic panniculitis, Mycosis fungoides like reaction, Follicular mucinosis, Malpighian epithelium, Porphyria Cutanea trade, and Pseudoporphyria, Exanthematous pustulosis, Sweet Syndrome, Panniculitis, SJS Rare cases - Small vessel vasculitis, Erythema nodosum, and GVHD of skin
Nilotinib	**Perifollicular, Hyperkeratotic, and Erythematous Maculopapular Eruptions**, Mild to moderate pruritus, Skin xerosis, Alopecia, Body hair loss, and Bullous Sweet syndrome
Bosutinib	Erythema, Maculopapular Eruption, Pruritic Rash, Allergic Dermatitis, Acne, Folliculitis, and Skin Exfoliation
Ponatinib	Erythematous and papular rashes **Skin xerosis**
Dasatinib	Macular, Papular or Exfoliative rash, Pruritus, Localized and generalized erythema, Papular Eruptions, Exfoliative rash, Pruritus, Hyperhidrosis, Alopecia, Xerosis, Acne, Urticaria, Dermatitis, Photosensitivity, Nail Disorders, and Pigmentary Changes, Skin Ulcers, Palmoplantar Erythrodysesthesia Syndrome. Rare Cases - Panniculitis, Acute Febrile Neutrophilic Dermatosis, And Bullous Disorders
Sorafenib	Stomatitis, Seborrheic dermatitis-like facial and scalp erythema, Subungual splinter hemorrhages, Periungual erythema, EM and EM-like eruptions, SJS, Eruptive melanocytic nevi and Drug-induced lentigines, Pyoderma gangrenosum, Generalized keratosis pilaris like eruption, Epidermal cysts, Nipple hyperkeratosis and/ or dysesthesia or dyskeratotic plaque with milia, New squamous cell carcinomas, Keratoacanthomas, and Inflammation of Preexisting Actinic Keratosis
Sunitinib	All grade hand-foot skin reactions, Seborrheic dermatitis-like facial and scalp erythema, Skin discoloration, Hair depigmentation, Facial edema, Transient scalp dysesthesia, Yellow skin pigmentation of the face Dry skin, Dermatitis, Stomatitis, Alopecia, Subungual splinter hemorrhages Phototoxicity, Pyoderma gangrenosum, Generalized keratosis pilaris-like eruption, Epidermal cysts, Nipple hyperkeratosis and/ or dysesthesia or Dyskeratotic plaque with milia
Erlotinib	Acneiform rash, Xeroderma, Pruritus, Paronychia
Cabozantinib	Hand-foot skin reaction, Generalized pigment dilution and/or Hair depigmentation, Xerosis, Scrotal erythema/ulceration, Subungual splinter hemorrhages
Vemurafenib	Dose-dependent papulopustular rash, Photosensitivity, Xerosis, Pruritus, Paronychia, Alopecia and hair follicle alterations, Hyperkeratosis, Pyogenic granulomas, Verrucous keratosis, Acantholytic dermatosis, Seborrheic keratosis, Verruca vulgaris, Hypertrophic actinic keratosis, Cutaneous squamous cell carcinoma and/ or Keratoacanthoma
Dabrafenib	Dose-dependent papulopustular rash, Xerosis, Pruritus, Paronychia, Alopecia and Hair follicle alterations, Hyperkeratosis, Pyogenic granulomas Verruca vulgaris, Hypertrophic actinic keratosis, Cutaneous squamous cell carcinoma and/ or Keratoacanthoma
Trametinib	Acneiform rash, Pruritus, Xerosis. Squamous cell carcinoma

### Rash

Rash is one of the commonest cutaneous manifestations of TKIs. Cutaneous manifestations resulting from EGFR inhibition affect multiple molecular pathways involved in cell growth and differentiation (Fig. 5 and Fig. 2) resulting in growth arrest, decreased migration, abnormal differentiation, and stimulation of inflammatory systems.^195^ Erlotinib is known to possess off-target inhibition of TK proteins and also explains the difference in the severity of lesions compared to gefitinib.^196^ Imatinib usually causes an erythematous, macular-papular rash a few days after initiation of treatment mediated through direct pharmacologic toxicity and is usually self-limited.^197^ Additionally, inhibition of KIT, VEGFR, and PDGFR may play a similar role.^193^ Use of emollients, and in case of infection, topical antibiotics for 2 weeks are recommended. Topical corticosteroids and/or oral antibiotics can be added to the regimen for a few weeks. Dose reductions are sometimes needed for erlotinib and afatinib.^100^ *Acute generalized exanthematous pustulosis* and *neutrophilic dermatoses (Sweet syndrome)* were also identified in imatinib-treated CML patients.^198,199^ BTKI therapy exhibited asymptomatic nonpalpable petechial rash, palpable rash, and erythema nodosum. The nonpalpable petechial rash can be explained by underlying ibrutinib-induced platelet dysfunction. They respond well to dose reduction and steroids.^168,200,201^

### Stevens–Johnson syndrome (SJS)

Numerous cases of SJS have been reported within 1 week of imatinib-treated CML patients.^202^ Imatinib desensitization along with prednisone is recommended for the initial management of severe mucocutaneous eruptions.^203^ However, newer alternatives like dasatinib and nilotinib have largely avoided this need.^197^ Similar reports have been published with the use of vandetanib.^154^

## Metabolic dysfunctions

### Glucose metabolism

Increasing evidence suggests an association between imatinib, sunitinib, and nilotinib and glucose metabolism. In the adjuvant GIST trial, hyperglycemia was demonstrated in patients on imatinib therapy. Imatinib was associated with the severity of hypoglycemia in GIST patients.^204^ Studies showed enhancement of B-cell survival by imatinib, potentially contributing to the observed hypoglycemic effects.^205^ Sunitinib is associated with the incidence of hypoglycemia in 15–19% of patients who present mostly 4 weeks after treatment.^206^ Regression of pancreatic islets, modulation of IGF-1 (insulin-like growth factor 1) signaling, and decreased glucose uptake are the proposed hypotheses. In diabetic patients on TKIs, assessment of glycemic control is recommended. In a nondiabetic patient on TKIs, periodic monitoring of HbA1c and blood glucose levels are recommended.^207^

### Fluid retention

This is commonly reported after BCR-ABL TKI therapy, especially imatinib and to a lesser extent nilotinib, dasatinib, and bosutinib in CML patients.^173^ The most common edema locations associated with imatinib are the periorbital space and the lower limbs.^208^ Increased edema risk is reported in patients 65 years or older, and those with cardiac disease, renal insufficiency, HTN, hypercholesterolemia, and a history of autoimmune diseases.^15^ Regular monitoring of body weight, heart and lung symptoms, chest imaging, and peripheral tissue tone to detect edema is recommended. Edema is often reversible after treatment with diuretics and steroids. TKI cessation is required in severe cases, at least until the symptoms are controlled.^15^ Peripheral edema is a common side effect associated with MEK inhibitors. Mild cases involve treatment with compression therapy and head elevation after ruling out other causes such as renal failure and hypoalbuminemia.^179^

### Electrolyte imbalances

BCR-ABL TKIs are associated with electrolyte abnormalities (Table 1).^15^ Specifically, an increased incidence of early-onset, persistent hypophosphatemia is noted with imatinib, nilotinib, dasatinib, bosutinib, and ponatinib therapy. It is accompanied by increased phosphaturia with low-to-normal calcium levels, and secondary hyperparathyroidism.^209,210^ Therefore, recommendations for periodic monitoring and correction of electrolyte abnormalities in TKI therapy are of clinical significance.^15^

### Bone and mineral homeostasis

Studies are in progress to identify an association between imatinib and bone mineral density. However significant outcomes have yet to be determined.^167^ The proposed mechanisms include nonspecific inhibition of TKs expressed by osteoclasts and osteoblasts, including c-KIT and PDGFRA. Slowing of growth velocity and growth retardation was observed in children undergoing imatinib or dasatinib therapy for CML, a mechanism involving disturbances in the growth hormones/IGF-1 axis.^167,173^ Dasatinib leads to dysregulation of bone remodeling via osteoclast inhibition.^211^

## Endocrine dysfunctions

### Thyroid metabolism

Abnormal thyroid metabolism was reported with BCR-ABL TKIs such as imatinib, dasatinib, and nilotinib. Side effects are usually subclinical and transient, with no treatment necessary.^173^ Hypothyroidism was associated with imatinib, sunitinib, and sorafenib across several studies. Routine thyroid function tests are recommended before and after drug therapy, and after any changes in the TKI regimen. Overt hypothyroidism is treated with levothyroxine but TKI therapy can still be continued.^212^ Imatinib either worsens pre-existing hypothyroidism or causes de-novo hypothyroidism. Non-deiodination clearance has been described as the underlying mechanism of imatinib-associated hypothyroidism.^213^ A similar association is seen in studies with sunitinib used during therapy for gastrointestinal stromal cells and metastatic RCC. Sunitinib when given to a patient pre-treated with imatinib, showed higher TSH levels.^212^ Fatigue associated with sunitinib is possibly related to underlying hypothyroidism, which is an *off-target effect* of TKIs. Sorafenib is associated with a lower incidence of hypothyroidism when compared to sunitinib due to the difference in the degree of receptor inhibition.^214^ Serial monitoring of thyroid function tests at baseline and during TKI therapy is recommended. Symptomatic patients are often managed with thyroid hormone replacement with the continuation of cancer treatment.^212^

### Adrenal insufficiency

No overt suppression of the adrenal gland has been identified with sunitinib therapy. It is recommended to monitor adrenal functions in patients exposed to major stressors such as surgery, trauma, or severe infection.^215^

### Hypogonadism

Alterations in testosterone was reported in imatinib and dasatinib-treated patient with concomitant gynecomastia.^167^ Case reports showed that the development of painful gynecomastia with sunitinib therapy is due to its inhibition of the c-KIT/PDGFRA axis.^216^

## Pulmonary toxicity

### Pleural effusion

Pleural effusion is mostly seen with BCR-ABL TKIs with dasatinib having a high likelihood of pleural effusion as the late toxicity.^167^ This was reported in the DASISION-dasatinib trial which reported a 10% incidence of pleural effusion in the 1^st^ year, and 29% by the end of the study. Importantly, 20% of the patients had to discontinue dasatinib therapy due to toxicity.^217^ The incidence is higher in bosutinib in comparison to imatinib as per the BELA trial demonstrating discontinuation of bosutinib in 250 patients *vs*. no changes in imatinib.^218^ Dasatinib is associated with pulmonary toxicity in the form of unilateral or bilateral pleural effusions, and in some cases, it is associated with lung parenchymal infiltrates.^219,220^ An efficient strategy to reduce risk includes dose optimization.^221^ Management depends on the severity, and withdrawal of dasatinib in symptomatic patients until spontaneous resolution is recommended. Later, a lower dose of dasatinib or a switch to another TKI is recommended. Additional therapies may include a short course of steroids and thoracentesis in severe cases.^173^

### Interstitial lung disease (ILD)

ILD is a heterogeneous group of diseases ranging from mild radiographic lung infiltrates to fatal acute respiratory distress syndrome.^166^ ILD is associated with both EGFR TKIs and ALK TKIs. It has also been reported in ALK-TKIs such as crizotinib, ceritinib, and alectinib. The exact mechanism of ILD precipitated by EGFR TKIs is unknown but it is probably due to a decreased protective function of the EGF receptors localized on type 2 pneumocytes. The risk factors include a previous diagnosis of lung fibrosis, older age, poor performance status, male gender, smoking history, previous or concomitant ILD, comorbid pleural effusion, and coexisting heart disease.^65,166^ It is also the main cause of death in EGFR TKI therapy. If ILD is suspected, TKIs are discontinued. Once ILD is confirmed, the culprit agents should be stopped regardless of the severity.^166^

### Drug-induced pneumonitis

Pneumonitis is associated with several TKIs, including EGFR inhibitors^222^ and MEK inhibitors. In the case of pulmonary symptoms, a CT chest is performed. Once the infectious cause is excluded via bronchoalveolar lavage, a high dose of corticosteroids is indicated. Severe cases warrant permanent discontinuation of the culprit drugs with the reintroduction of BRAF inhibitors after remission, which is possible.^179^ Both capmatinib and tepotinib are associated with about 2–6% of drug-induced pneumonitis as per the GEOMETRY and VISION trials, respectively.^223–225^ Management involves permanent discontinuation of MET inhibitor along with steroid treatment.^226^ Switching with another TKI after the resolution of pneumonitis can be considered.^227^

## Neurological dysfunction

Clinical trials enrolling 23 CML patients treated with nilotinib therapy demonstrated an increased incidence of vascular adverse effects in response to an increase in TKI doses and duration of treatment leading to *cerebral infarction* (CI). CI is considered one of the fatal complications of treatment. The incidence of CI per 1000 person-years was 2.25 with imatinib, 6.47 with nilotinib, and 2.43 with dasatinib. The adjusted incidence rates of CI in the age and gender-matched Japanese general population was 3.342 per 1000 person-years.^228^ However, a large-scale population-based study in Sweden investigating vascular adverse effects in TKI therapy reported no increase in the CI incidence, though it was relatively higher with nilotinib.^86^ Another study from Korea demonstrated an increased incidence of stroke in nilotinib in comparison to dasatinib.^229^ This was further corroborated by the ENESTnd trial studying imatinib and dasatinib during a 5-year follow-up period.^87^ Studies also reported that males had a higher incidence compared to females.^228^ Imatinib is known to have a relatively safer vascular profile compared to other TKIs. Nilotinib is associated with an increased incidence of arterial occlusive disease as per a report by Hadzijusufovic et. al.^230^ In comparison, ponatinib showed a significant episode of serious cerebrovascular events.^231^. Fedratinib has been associated with cases of encephalopathy and MRI demonstrated findings consistent with Wernicke encephalopathy.^232^

### Pathogenesis

Nilotinib inhibits angiogenesis and endothelial cell proliferation leading to a slow blood flow recovery rate post-ischemia. It also promotes the expression of various pro-atherogenic cytoadhesion molecules.^233^ Nilotinib demonstrated an imbalance between pro/anti-inflammatory processes, leading to a hypothesis that the pro-inflammatory state activates pro-atherothrombosis via enhanced lipid peroxidation. Genetic pro-atherothrombotic predisposition conferred by LOX-1 may play an important role.^234^ These changes were absent in imatinib. Ponatinib inhibits neo-angiogenesis of vascular endothelial cells. However, more trials are necessary to study the vascular adverse effects of ponatinib and dasatinib.^235^

### Management

Neurovascular risk scores help with patient selection requiring closer monitoring and detection of early signs. Periodic lipid profiling and blood glucose examinations, BP monitoring, Doppler ultrasound of the selected blood vessels, and intimal medial thickness measurement have been employed in cases to monitor TKI side effects.^236^ Decisions regarding therapy modifications should be based on the severity of the vascular event(s), the response state of CML, and the availability of alternative treatment options for CML.^231^ In the case of mild cases, a dose reduction with close monitoring has been recommended. It is evidenced by data that there is a reduction in the risk of arterial thrombotic events by about 40% when the ponatinib dose is reduced by 15 mg.^237^

## Renal toxicity

Although uncommon, renal dysfunction has been associated with TKI therapy, particularly reported with imatinib and bosutinib (Table 1). Imatinib has shown a decline in GFR and new-onset CKD in 22% of patients. Renal toxicity is also reported in <10% of patients taking bosutinib.^167^ TKIs, specifically VEGFIs, are associated with the development of *proteinuria*. It has been predominantly seen with sunitinib, sorafenib, and pazopanib therapies. Further, sorafenib and sunitinib manifest as albuminuria, thrombotic microangiopathy, and acute interstitial nephritis.^154^ Sorafenib requires dose adjustments for creatinine clearance less than 40 mL/min.^186^ Cediranib, a newer potent VEGF inhibitor, has shown proteinuria in 30% of patients within 2 weeks of therapy. Long-term data are still unavailable.^238^

The underlying mechanisms are suggested to be due to endothelial damage by TKIs resulting in local thrombosis (Fig. 5). The resultant thrombotic microangiopathy manifests as low-grade proteinuria with no systemic findings. Renal biopsy is performed only in selected patients with high-grade proteinuria.^238^ Since the risk of proteinuria is associated with the progression of renal disease, measures must be taken to screen these patients using baseline urinalysis and protein-to-creatinine ratio before starting TKIs. A urine protein to creatinine ratio of ≥ 1 or 24-hour urine with ≥ 1 gr/dL/24 hours of protein should prompt intervention.^154^ In the case of severe and persistent proteinuria when thrombotic microangiopathy is evident through renal biopsy, the benefits *vs*. risks of continuation or TKI therapy modifications should be considered. This is particularly difficult in conditions where TKIs are the last line of therapy for advanced cancer. Proteinuria is often accompanied by HTN. Thus, ACEIs or ARBs are generally viable treatments. Diuretics, CCBs, or BBs can be used, although benefits are yet to be demonstrated.^238^

Acute renal failure or electrolyte disorders were reported in combination therapy of BRAF and MEK inhibitors with an increase in serum creatinine.^239,240^ Pathology includes acute on chronic tubular interstitial damage.^241,242^ Drug cessation with reintroduction at a lower dose is done in higher-grade kidney injuries. Rehydration may be beneficial in a few patients with pre-renal failure.^179^

## Ocular reactions

### Ocular inflammation (uveitis, conjunctivitis)

These side effects are rarely seen with BRAF inhibitors. Mild cases are treated with topical steroids. Discontinuation or dose reduction with severe episodes is recommended.^243^

*Retinal vein occlusion* is observed with both BRAF inhibitor monotherapy or a combination of BRAF and MEK inhibitor therapy.^243^ Since the frequency is similar to patients not on therapy, the causal relationship is still up for debate. It is generally recommended to discontinue therapy with the constitution of ophthalmologic therapy.^244^

*Central serous chorioretinopathy* is an adverse effect with fluid accumulation in the retina and is associated with a combination of BRAF and MEK inhibitor therapies.^245^ It is seen in about 25% of patients treated with erdafitinib, an FGFR inhibitor used to treat metastatic urothelial carcinoma. This is often asymptomatic but can rarely lead to transient visual disturbances. It is usually bilateral, multifocal occurring within hours, days, or weeks of initiation and for the most part, attributed to the MEK inhibitors. The contributory mechanism is not completely understood. It requires frequent monitoring^246^ and can be diagnosed with optical coherence tomography.^245^ Usually, no treatment is required. However, discontinuation or reduction of MEK inhibitors with severe manifestations can be considered.^179^

## Opportunistic infections

These are seen in >50% of patients taking BTK TKIs. The patients receiving these TKIs are immunocompromised and are at increased risk for infections. It is most likely to occur in patients with relapsed or refractory type cancers.^247^ It is hypothesized that irreversible^248^ inhibition of ITK52^249^ and impairment of macrophages increase susceptibility to infection.^250,251^

### Pneumonia

This is the most common infective complication and the major contributor to death, in about 2% of patients.^248^ Pneumonia was reported in about 12% of patients with ibrutinib therapy. Aspergillus fumigatus and Pneumocystis jirovecii (PJP) have been isolated as the culprit organisms.^168^ Other organisms included Histoplasma, Cryptococcus, and Nocardia.^248^ In high-risk patients or those with a previous history of infections, PJP prophylaxis should be considered.^252^ In the case of Aspergillus fumigatus, a high incidence of infection has been reported early in treatment with higher rates in patients receiving steroids.^168^ There are differing opinions regarding management which dictates cessation of TKIs in acute infection until a definitive diagnosis has been established or during the grade 4 infection.^253,254^ Nevertheless, therapy can be restarted on improvement as per the healthcare provider. The administration of vaccines against influenza and pneumococcus has been advocated before the initiation of therapy along with the addition of recombinant, adjuvant vaccine against varicella-zoster virus.^255^ Intravenous immunoglobulin therapy should be considered in patients with recurrent infections or for known hypogammaglobulinemia.^168^

### Invasive Aspergillosis

Combination drug studies in primary CNS lymphoma (PCNSL) reported invasive aspergillosis infections in 39% of patients. Single-agent studies demonstrated aspergillosis in 5–11% of patients.^248^

### Urinary tract infections (UTIs)

These were the most common infections found in fedratinib-treated patients.^232^

## Hematological reactions

### Cytopenias

These side effects include anemia, leucopenia, neutropenia, and thrombocytopenia most commonly identified in CML patients being treated with *BTK TKIs*.^256^ Cytopenias were often reported at the initiation of therapy.^15^ Nilotinib or dasatinib are associated with an increased incidence of cytopenias. However, when the second-generation drugs were given as first-line therapy for CML rates of cytopenias were lower for both drugs. Nilotinib is associated with neutropenia in 4–12% and thrombocytopenia in 2–12% of patients. Similarly, dasatinib is associated with neutropenia in 21% and thrombocytopenia in 10–19% of patients.^15^ In the ENESTnd and DASISION trials, nilotinib and dasatinib were demonstrated to be less hemotoxic than imatinib.^168^ According to a meta-analysis including 17 studies, dasatinib appeared to be the most hemotoxic drug used in CML compared to nilotinib which was the safest.^256^ Ruxolitinib has been associated with dose-dependent anemia, high-grade neutropenia, and dose-limiting thrombocytopenia.^257^ Fedratinib is frequently associated with anemia during treatment.^232^

### Mechanisms of cytopenia

Anemia is due to the inhibition of c-KIT by TKIs.^258^ Leukopenia and neutropenia can be due to the binding of drugs to several key kinase targets of the immune system, such as Lck, Lyn, Btk, and Src.^259^ Cytopenia in BCR-ABL TKIs is likely due to decreased levels of healthy residual Philadelphia chromosome-negative bone marrow being unable to reconstitute peripheral blood counts rather than direct toxicity of the drugs towards normal hematopoietic cells.^260^ Additionally, dasatinib has inhibitory effects against platelets which are rapidly reversible with the interruption of therapy.^261,262^

### Management of cytopenia

Most cases are mild to moderate severity cytopenias which are self-limited. Severe manifestations are carefully observed necessitating monitoring of blood counts weekly in the first month and monthly thereafter for the next 3 months.^263^ Supportive treatment with changes in dosage or temporary cessations of TKI is undertaken.^264^ Supportive treatments include recombinant erythropoietin or granulocyte-stimulating factor and thrombopoietin receptor agonists. Additional immunosuppressive therapies such as short-course corticosteroids and anti-CD20 monoclonal antibodies can be considered.^173^

### Bleeding risk

BTK TKIs are most commonly associated with bruising in two-thirds of patients with no increased risk of major bleeding. Major bleeding occurs rarely in about 2–9% of patients.^168^ In an integrated analysis, ibrutinib was associated with an increased risk of bleeding of 4.4% compared to the test drug with 2.8%. Additionally, both groups experienced increased use of anticoagulants or antiplatelet agents.^265^ On the other hand, acalabrutinib in the ELEVATE-TN trial reported major bleeding in 2% of patients and minor bleeding in 37% of patients.^266^ Zanubrutinib is more selective in its BTK inhibition with no effects on platelet function or receptor shedding compared to ibrutinib.^267^ Dasatinib and ponatinib can cause platelet dysfunction, potentially increasing the risk of bleeding.^268^ As per a combined report of 4 multi-center studies dasatinib use resulted in grade 3 or 4 bleeding in up to 10% of patients.^269^ Dasatinib also causes thrombocytopenia by impairing megakaryocyte formation.^270^ This is of particular importance in the application of anticoagulation and anti-platelet drugs in vascular disease in ponatinib and dasatinib-treated patients.^231^ Mild mucosal bleeding can occur from inhibition of VEGFR-2 leading to microvascular leaks from endothelial cell damage.^154^

BTK TKIs can be held for 3 days or 7 days before and after minor and major invasive procedures respectively due to the increased risk of periprocedural bleeding.^271^ For minor bleeds, temporary cessation of therapy is done until resolution. In severe grade ≥3 bleeding, platelet transfusion irrespective of platelet count is considered to overcome bleeding.^272^ Concurrent use of drugs with increased bleeding risk is avoided. Alternative CLL therapy agents are considered if the patient requires dual antiplatelet therapy.^273^

## Miscellaneous side effects

### Headache

This symptom is most commonly associated with the second-generation BTK TKIs, acalabrutinib. Grade 1 or 2 headaches were mostly reported in 70% of patients during the first few weeks of therapy.^266,274^ Comparatively, in ibrutinib, this symptom presents at a later stage of treatment. Symptomatic relief can be achieved with extended treatment and the administration of acetaminophen with or without caffeine.^168^

### Secondary malignancies

Case reports published from a review of over 9000 CML patients showed no clear increase in any malignancies, though it has to be mentioned that the patients had a relatively short follow-up.^275^ Similarly, a single large institutional study with a follow-up of 107 months reported no second cancers.^276^ On the contrary, a small study of 52 Japanese patients and a Swedish registry study with 868 patients suggested an increased risk of malignancies over the general population.^277^

### TKI and pregnancy

Limited data are available on the safety of TKIs in pregnancy. TKI’s effects on fetal growth and development are not known. Current guidelines suggest avoiding pregnancy while undergoing therapy.^278^ Some studies suggest that imatinib is associated with fetal malformations such as low birth weight, whereas erlotinib and lapatinib can cause oligohydramnios. Therefore, close follow-up of growth through ultrasonography and amniotic fluid index is recommended in these patients.^279^ In individuals who desire pregnancy, a 3-month washout period of TKI therapy is recommended before conception.^280^ In the case of TKI therapy being necessary during pregnancy, interferons may be used safely. However, its toxicity and slow response time should be considered.^281^ An alternate therapy with hydroxyurea is also safe during pregnancy, given in pulses to avoid high white blood cell counts.^282^

## Drug–drug interactions

### Cytochrome P450 metabolism

Sorafenib, regorafenib, lenvatinib, and cabozantinib are metabolized by the liver enzyme CYP3A4, and as such concurrent use of drugs modulating the activity of CYP3A4 can affect tolerability.^283^ Thus, the administration of a potent CYP3A4 inhibitor, antifungal ketoconazole can cause increased plasma levels. Thus, CYP3A4 inhibitors should be avoided with regorafenib and cabozantinib. Similarly, CYP3A4 inducers have the potential to reduce plasma TKI levels and decrease efficacy.^284^

### P-glycoprotein interactions

P-glycoprotein (P-gp) is inhibited by sorafenib and cabozantinib leading to the accumulation of P-gp substrates. Thus measures should be undertaken to avoid these drugs in patients receiving sorafenib or cabozantinib.^285,286^ Furthermore, metabolites of regorafenib which are P-gp substrates should therefore be avoided with P-gp inhibitors or inducers.^228^

## Future perspectives

The development of small molecule TKIS for cancer therapies has been growing at a rapid pace. Despite significant progress in cancer care, the utility of TKIs still faces significant challenges in terms of their tissue selectivity, efficacy, adverse events, and drug resistance. In addition to TKIs, there are several new categories of cancer-targeted drug discovery in development. For example, transcript-targeted therapies based on the use of RNA interference (RNAi) and antisense oligonucleotides (ASOs),^287^ and other RNA-based therapeutics such as Apatorsen, Cenersen, Custirsen^288^ for use in cancer. Targeting transcription and translation may affect multiple pathways, rather than the targeted one, and there are inevitable side effects arising from the fact that all cells require transcription and translation for their proper function. Similarly, humanized antibodies such as Pembrolizumab (PD-1antibody) or a combination of TKIs and PD-1 blockers are gaining extensive popularity in cancer care.^289–291^ The antibodies can induce immune-related and other side effects affecting multiple organ systems. Others include antibody-drug conjugate (ADC) drugs such as polatuzumab vedotin-piiq (Polivy), enfortumab vedotin-ejfv (Padcev), that are either already approved by the FDA or are in the development phase.^292–294^ Each of these new classes of drugs carries a separate set of challenges and can cause complex cardiac and extracardiac complications that would require in-depth knowledge and expertise to manage such complications in cancer patients.

When drug-associated toxicity is identified, it is imperative to understand the precise mechanisms of specific organ toxicity and to identify patients at risk for such events. A personalized approach must be incorporated in toxicity assessment in drug selection.^295^ Future studies should focus on the utilization of targeted therapies using personalized pharmacogenomics tools. This approach will address the variability of drug responses in individual patients, resulting in the reduction of adverse events.^296^ The combination of TKIs with *nanoscaled transporters* is another promising approach, resulting in the prevention of bystander adverse effects. Nanomedicine has many other advantages beyond the reduction of toxic effects to improve patient outcomes.^297^ The main objective is to facilitate specific drug biodistribution to the target areas in an adequate local concentration with minimal loss of volume or activity in blood. It efficiently reduces the toxicity towards healthy cells and tissues, as well as avoids the development of drug resistance.^298^
